# All-cause and cause-specific mortality among people with severe mental illness in Brazil's public health system, 2000–15: a retrospective study

**DOI:** 10.1016/S2215-0366(22)00237-1

**Published:** 2022-10

**Authors:** Ana Paula Souto Melo, Ilse N Dippenaar, Sarah Charlotte Johnson, Nicole Davis Weaver, Francisco de Assis Acurcio, Deborah Carvalho Malta, Antônio Luiz P Ribeiro, Augusto Afonso Guerra Júnior, Eve E Wool, Mohsen Naghavi, Mariangela Leal Cherchiglia

**Affiliations:** a Universidade Federal de São João Del Rei, São João del-Rei, Brazil; b Universidade Federal de Minas Gerais, Belo Horizonte, Brazil; c Institute for Health Metrics and Evaluation, University of Washington, Seattle, WA, USA; d Department of Health Metrics Sciences, University of Washington, Seattle, WA, USA; e Centro Colaborador do SUS para Avaliação de Tecnologias e Excelência em Saúde, Belo Horizonte, Brazil; f Telehealth Center and Cardiology Service, Hospital das Clínicas, and Department of Internal Medicine, Faculdade de Medicina, Belo Horizonte, Brazil

## Abstract

**Background:**

People with severe mental illness have a mortality rate higher than the general population, living an average of 10–20 years less. Most studies of mortality among people with severe mental illness have occurred in high-income countries (HICs). We aimed to estimate all-cause and cause-specific relative risk (RR) and excess mortality rate (EMR) in a nationwide cohort of inpatients with severe mental illness compared with inpatients without severe mental illness in a middle income country, Brazil.

**Methods:**

This national retrospective cohort study included all patients hospitalised through the Brazilian Public Health System (Sistema Único de Saúde [SUS]-Brazil) between Jan 1, 2000, and April 21, 2015. Probabilistic and deterministic record linkages integrated data from the Hospital Information System (Sistema de informações Hospitalares) and the National Mortality System (Sistema de Informação sobre Mortalidade). Follow-up duration was measured from the date of the patients’ first hospitalisation until their death, or until April 21, 2015. Severe mental illness was defined as schizophrenia, bipolar disorder, or depressive disorder by ICD-10 codes used for the admission. RR and EMR were calculated with 95% CIs, comparing mortality among patients with severe mental illness with those with other diagnoses for patients aged 15 years and older. We redistributed deaths using the Global Burden of Diseases, Injuries, and Risk Factors Study methodology if ill-defined causes of death were stated as an underlying cause.

**Findings:**

From Jan 1, 2000, to April 21, 2015, 72 021 918 patients (31 510 035 [43·8%] recorded as male and 40 974 426 [56·9%] recorded as female; mean age 41·1 (SD 23·8) years) were admitted to hospital, with 749 720 patients (372 458 [49·7%] recorded as male and 378 670 [50·5%] as female) with severe mental illness. 5 102 055 patient deaths (2 862 383 [56·1%] recorded as male and 2 314 781 [45·4%] as female) and 67 485 deaths in patients with severe mental illness (39 099 [57·9%] recorded as male and 28 534 [42·3%] as female) were registered. The RR for all-cause mortality in patients with severe mental illness was 1·27 (95% CI 1·27–1·28) and the EMR was 2·52 (2·44–2·61) compared with non-psychiatric inpatients during the follow-up period. The all-cause RR was higher for females and for younger age groups; however, EMR was higher in those aged 30–59 years. The RR and EMR varied across the leading causes of death, sex, and age groups. We identified injuries (suicide, interpersonal violence, and road injuries) and cardiovascular disease (ischaemic heart disease) as having the highest EMR among those with severe mental illness. Data on ethnicity were not available.

**Interpretation:**

In contrast to studies from HICs, inpatients with severe mental illness in Brazil had high RR for idiopathic epilepsy, tuberculosis, HIV, and acute hepatitis, and no significant difference in mortality from cancer compared with inpatients without severe mental illness. These identified causes should be addressed as a priority to maximise mortality prevention among people with severe mental illness, especially in a middle-income country like Brazil that has low investment in mental health.

**Funding:**

Bill and Melinda Gates Foundation, Fundação de Amparo a Pesquisa do Estado de Minas Gerais, FAPEMIG, and the Coordenação de Aperfeiçoamento de Pessoal de Nível Superior—Brasil.

## Introduction

Life expectancy among those with severe mental illness—including schizophrenia, bipolar and depressive disorders, and their related spectrum disorders—is 10–20 years shorter than the general population.^1^, ^2^, ^3^, ^4^ Life expectancy for those with severe mental illness is around 15 years lower for women and 20 years lower for men compared with the general population.^3^ Excess mortality is not solely explained by the increased risk of death by suicide and other injuries; communicable and non-communicable diseases (NCDs) account for the majority of premature mortality.^5^, ^6^, ^7^ Among NCDs, cardiovascular disease is the leading preventable cause of early deaths in those with severe mental illness.^5^ Researchers have discussed the necessity of efforts to increase timely and effective health-care interventions, with the aim of reducing overall mortality in this vulnerable population.^8^, ^9^ However, there have been various barriers to scaling up evidence-based care and improving the life expectancy of these patients, especially in Brazil, a middle-income country with low levels of investment in mental health.^10^

**Research in context**

**Evidence before this study**

We searched MEDLINE, PsycINFO, and EMBASE with the following search terms: “mental disorders”, “serious mental illness”, “severe mental illness”, “schizophrenia”, “depression”, “bipolar disorder”, and “mortality”. We searched for studies published from from Jan 1, 1980, to March 31, 2022, with no language restrictions. Previous studies indicated a high risk of mortality among people with severe mental disorders; however, most of these studies were done in high-income countries (HICs), particularly in western Europe. Meta-analyses of all-cause mortality showed that the pooled relative risk (RR) of mortality among those with mental ill health was 2·22 (95% CI 2·12–2·30) compared with the general population of people without mental disorders, with an RR of 2·54 (2·35–2·75) for psychoses and 1·86 (1·73–2·00) for mood disorders. Risks vary across the life course and over time, and among diagnoses, sexes, and treatment sites. Follow-up duration ranged from 1 year to 52 years, with a median of 10 years. Previous studies included sample sizes that ranged from 22 individuals to 664 094 individuals. Most studies included samples of inpatients or inpatients and outpatients. There is a persistent mortality gap for people with severe mental disorders that do not improve over time. Few data exist on broader cause-specific mortality and from low-income and middle-income countries. Previous studies in HICs did not find an increased risk of mortality for people with severe mental disorders for some causes, such as idiopathic epilepsy, tuberculosis, and acute hepatitis, nor a high risk of mortality for younger populations.

**Added value of this study**

We found that Brazil, a middle-income country, had a high mortality among people with severe mental illness, and the pattern of mortality was distinct for some conditions compared with HICs. Our findings showed a systemic excess mortality and significantly higher levels of injuries (such as homicide), infections (such as tuberculosis, acute hepatitis, and HIV), and chronic diseases (such as epilepsy), with a particularly high risk of ischaemic heart disease in people with severe mental disorders. We did not find an excess mortality associated with cancer in inpatients with severe mental illness. Individuals younger than 60 years were at higher risk of mortality, especially those younger than 30 years. We evaluated all significant RRs and excess mortality ratios and detailed the leading causes of death by sex and age group, with a follow-up of 15 years. The data indicated that during the follow-up period younger people had a greater risk of death, and females had a higher RR than did males. Mortality from cardiovascular diseases in people with severe mental illness contributes to more deaths overall compared with other causes (except for death by suicide) because of the high prevalence. We found the lowest RR for deaths in older adults (≥60 years).

**Implications of all the available evidence**

As in HICs, it is important for middle-income countries to address the high prevalence of communicable and non-communicable diseases, especially cardiovascular disease, and injuries in people with severe mental illness, particularly those 60 years and younger.

Brazil has undergone an epidemiological transition largely attributable to declines in communicable diseases alongside an increasing NCD burden, with large health inequities observed between different regions of the country. Ischaemic heart disease was the leading cause of death in Brazil in 2019.^11^ In 2015, mental disorders accounted for 9·5% of all disability-adjusted life-years (DALYs), ranking in the third and first position for DALYs and years lived with disability, respectively. Brazil has a public health system (the Sistema Único de Saúde [SUS]) that guarantees the right to universal health care for all citizens. 71·5% of Brazilian people exclusively use the SUS for health care, with the remaining 28·5% of the population having access to private health insurance.^12^ The SUS has diverse health-care services, with a priority on primary health services. Brazil has been reducing beds in psychiatric hospitals and replacing them with a community-based system. The rate of hospitalised psychiatric patients in Brazil decreased from 188·5 per 100 000 inhabitants in 2000 to 94·4 per 100 000 inhabitants in 2014.^13^

Premature mortality in psychiatric patients has been well described.^14^, ^15^, ^16^, ^17^, ^18^ However, there is a paucity of research from low-income and middle-income countries (LMICs).^19^ Even in high-income countries (HICs), hospitalisations are not always linked with mortality, hindering the quantification of excess mortality.^20^ Additionally, there is a gap in the literature when it comes to long periods of follow-up. In this paper, we estimate all-cause and cause-specific relative risk (RR) and excess mortality rate (EMR) in a nationwide cohort of inpatients with severe mental illness compared with other inpatients from 2000 to 2015 in Brazil, using a database from the Hospital Information System (Sistema de informações Hospitalares [SIH]) that was linked with the Mortality Information System (Sistema de Informação sobre Mortalidade [SIM]).^21^

## Methods

### Study design and participants

In this retrospective study, we identified a national longitudinal cohort of patients that integrated health data from the public health system (SUS), including all 27 Brazilian states from Jan 1, 2000, to April 21, 2015. We compared inpatients with severe mental illness with inpatients with other diagnoses. We used the following diagnoses according to the ICD-10 to indicate the presence of severe mental illness: schizophrenia and schizoaffective disorder (F20–F20.9, F25–F25.9), bipolar disorder (F30–F31.9, F34.0), and depressive disorders (F32–F33.9, F34.1). We disregarded multiple diagnoses of severe mental illness, considering only the earliest observed diagnosis for each individual. Dates of death and cause of death information for the cohort were derived from a linkage database. We identified a set of simple patient and admission inclusion criteria that were used to subset our data and to counteract the known false positive linkage rate. For example, we excluded linked patients recorded with multiple birth years or multiple death records. To ensure a consistent control group and avoid inflating our study with otherwise healthy patients, we excluded any obstetrics-related admissions. There were no further limitations on patient inclusion in our analysis. We mapped all ICD-10 diagnosis codes to a cause list for easier analysis, using separate maps for underlying causes of death and non-fatal diagnoses. We classified codes assigned to the underlying cause of death as garbage codes, which are either not specific enough, an immediate or intermediate cause of death, or an impossible cause of death. Approximately 23·5% of all deaths were assigned to garbage codes (table 1). Following The Global Burden of Diseases, Injuries, and Risk Factors (GBD) Study methodology, we performed garbage code redistribution for any garbage-coded deaths.^22^ If a garbage code was assigned as an underlying cause, then fractions of this death would be assigned to each of the target causes in the redistribution process, since each death in the GBD could be allocated to only one underlying cause as per ICD categorisation of causes of death. See the appendix (p 4) for our definitions of garbage codes. Before any analysis began, the data were deidentified, preserving the patients’ privacy. The study was approved by the Research Ethics Committee of the Federal University of Minas Gerais (CAAE 44121315.2.0000.5149). The GBD Study uses de-identified data; the waiver of informed consent was reviewed and approved by the University of Washington Institutional Review Board (study number 9060).

**Table 1:** Summary statistics of the dataset by age and sex

	**Total population**	**Sex**		**Age (years)**		
		Male	Female	15–29	30–59	≥60
**All patients**						
Population	72 021 918	31 510 035	40 974 426	19 037 302	25 545 504	11 974 658
Deaths	5 102 055	2 862 383	2 314 781	289 652	1 655 103	3 222 086
Garbage-coded deaths	1 198 436 (23·5% of total deaths)	649 055	567 175	48 907	349 240	809 719
**Patients with severe mental illness**						
Population	749 720	372 458	378 670	199 550	495 776	57 006
Deaths	67 485	39 099	28 534	8396	45 356	14 282
Garbage-coded deaths	18 405 (27·3% of total deaths)	10 556	7897	2114	12 745	3690
**Patients with depressive disorders**						
Population	169 319	61 530	107 789	39 290	112 823	15 254
Deaths	14 665	7523	7142	1324	9107	4200
**Patients with bipolar disorders**						
Population	199 561	68 793	130 768	48 865	135 535	14 175
Deaths	15 802	6629	9173	1574	10 726	3485
**Patients with schizophrenia and schizoaffective disorders**						
Population	462 630	273 228	189 402	128 609	300 786	31 365
Deaths	45 487	28 712	16 775	6310	31 341	7793

A patient might appear in more than one age or sex category if their demographic information changed during the observation period. However, patients were never double counted in aggregate groups. See the appendix (pp 3–4) for information on our case definitions and our use of garbage codes.

Our study follows the Guidelines for Accurate and Transparent Health Estimates Reporting (GATHER). The full GATHER checklist is provided in the appendix (pp 47–48).

### Data sources

This study integrated health data from the public health system (SUS), which covers a population of 150 million inhabitants who are dependent on the SUS in Brazil.^12^ We used data from 72 021 918 patients admitted to SUS hospitals from Jan 1, 2000, to April 21, 2015, the period of the contracted data agreement. In this period, the annual number of hospital admissions in the SUS was around 11 million and the mean annual expenditure on these hospitalisations was about US$3·89 billion.^23^ Data were obtained for this period from the National Health Database, a population-based national cohort developed through probabilistic and deterministic record linkage of data from the following SUS information systems: the Hospital Information System (SIH) and the Mortality Information System (SIM).^21^

Hospital data from the SIH, an administrative database that contains information about hospital admissions, covered all inpatient procedures and hospital discharges, transfers, and deaths in the public health system in Brazil. Data included dates of admission and discharge, primary and secondary diagnosis codes (coded using the ICD-10), and codes for procedures done during the hospital stay.^21^ Mortality data (from the SIM) included the underlying cause of death and secondary contributing causes (coded using the ICD-10) and date of death.^21^ As noted previously, the GBD Study uses de-identified data and personal identifying details included in the underlying data were not included in the analysis.

### Statistical analysis

We did subgroup analyses on sex and age using age bins 15–29, 30–59, and ≥60 years. For each group and underlying cause of death considered, we constructed a 2 × 2 contingency table to count the mortality outcomes for all patients in the group. To study the longer-term impacts of severe mental illness, we examined mortality outcomes at least 3 months after a patient's first severe mental illness diagnosis. We then calculated RR and EMR for each of the contingency tables to quantify both the relative and absolute cause-specific disease burden of severe mental illness. To analyse the trend of severe mental illness-related excess mortality over time, we selected five additional follow-up periods (3 months to 1 year, 1–2 years, 2–5 years, 5–10 years, and >10 years after initial severe mental illness diagnosis) and did an identical analysis using each of these time windows. See the appendix (pp 3–7) for additional details on the data processing steps and definitions of RR and EMR. All analyses were done in Python version 3.9.

### Role of the funding source

The funders of the study had no role in study design, data collection, data analysis, data interpretation, or writing of the report.

## Results

From Jan 1, 2000, to April 21, 2015, 72 021 918 patients (31 510 035 [43·8%] recorded as male and 40 974 426 [56·9%] recorded as female; mean age 41·1 [SD 23·8] years) meeting the inclusion criteria were admitted to the Public Health System (SUS) hospitals, covering all 27 Brazilian states, with 749 720 patients (372 458 [49·7%] recorded as male and 378 670 [50·5%] as female) admitted to hospital with severe mental illness. Among the 5 102 055 deaths registered (2 862 383 [56·1%] recorded as male and 2 314 781 [45·4%] as female) during the study period, 67 485 occurred among patients with severe mental illness (39 099 [57·9%] recorded as male and 28 534 [42·3%] as female; (table 1). Among the total deaths, we redistributed 1 198 436 (23·5%) deaths that were garbage-coded using the methodology of the GBD Study (appendix p 4). The proportion of the deaths registered as garbage codes decreased from 41·3% in 2000 to 33·3% in 2015 for mortality data in Brazil.

The RR for mortality in inpatients with severe mental illness compared with inpatients without severe mental illness during the follow-up period longer than 3 months, and considering the earliest observed diagnosis at admission, was 1·27 (95% CI 1·27–1·28; table 2). Overall, among patients with severe mental illness, the RR of mortality for females was higher than that for males. Among age groups, we found a higher RR for younger ages (15–29 years) followed by those aged 30–59 years, and no significant increased risk in older adults (table 2).

**Table 2:** Relative risk and excess mortality rate by age and sex

	**Total**	**Sex**		**Age (years)**		
		Male	Female	15–29	30–59	≥60
Relative risk	1·27 (1·27–1·28)	1·16 (1·15–1·17)	1·34 (1·32–1·35)	2·82 (2·76–2·88)	1·42 (1·41–1·44)	Not significant
Excess mortality rate	2·52	2·16	2·17	3·50	6·85	Not significant

All patients represented here have had severe mental illness for longer than 3 months

**Figure 1:**
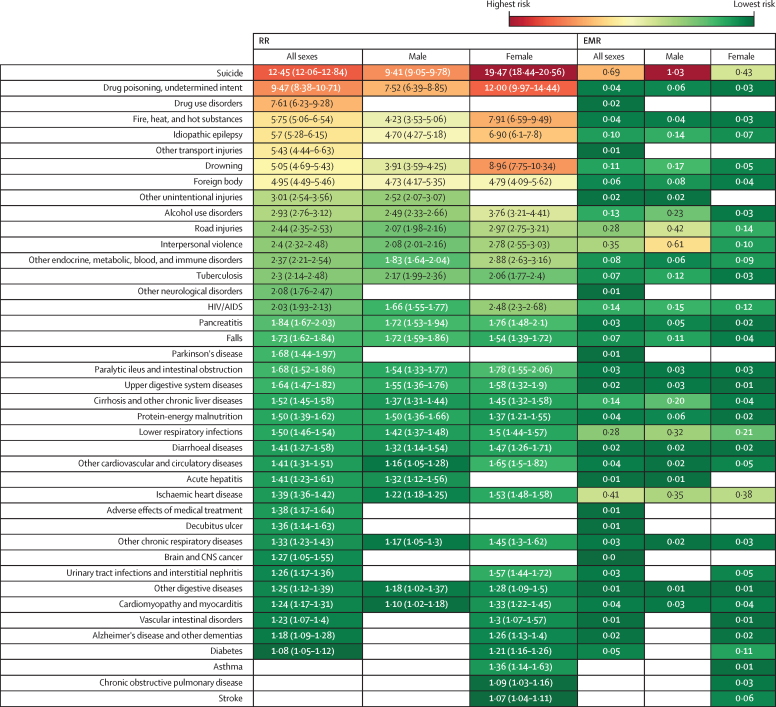
Significant RR of mortality and EMR for all ages by sex with severe mental illness exposure longer than 3 months Blank cells indicate non-significance. RR=relative risk. EMR=excess mortality rate.

**Figure 2:**
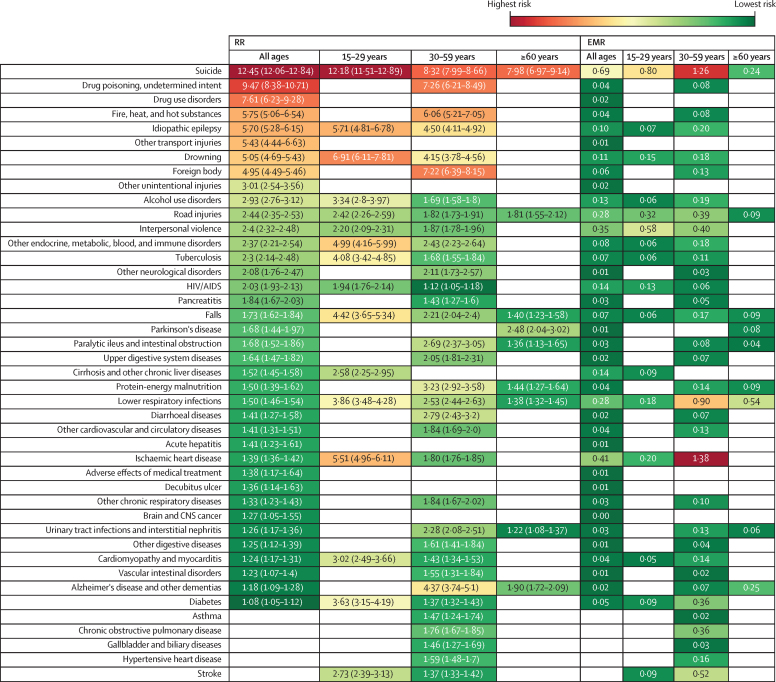
Significant RR of mortality and EMR for all sexes by age with severe mental illness exposure longer than 3 months Blank cells indicate non-significance. RR=relative risk. EMR=excess mortality rate.

We found an EMR of 2·52 in inpatients with severe mental illness compared with inpatients who did not have severe mental illness. For most conditions, the EMR was higher for males than for females (figure 1) and higher in those aged 30–59 years compared with other age groups (table 2; figure 2).

Injuries accounted for 19·9% of all deaths in the population with severe mental illness, including unintentional causes such as fires, drowning, foreign body, road injuries, and falls (figure 1).

The RR for unspecified drug poisoning of undetermined intent (overdoses) was higher in females (12·0, 95% CI 9·97–14·44) than in males (7·52, 6·39–8·85). Suicide accounted for 32·4% of deaths by injury. Suicide was associated with the highest RR and EMR in both sexes and all age groups (Figure 1, Figure 2). The RR for suicide was higher in females than in males (19·47, 95% CI 18·44–20·56 *vs* 9·41, 9·05–9·78). Among different age groups, the highest risk of suicide was found in those aged 15–29 years. Males and those aged 30–59 years had the highest EMR from suicide. Additionally, the RR for homicide for females was 2·78 (2·55–3·03) and for males was 2·08 (2·01–2·16; Figure 1, Figure 2).

Communicable diseases accounted for 13.6% of all deaths in the population with severe mental illness. HIV had an RR of 2·03 (95% CI 1·93–2·13) and EMR of 0·14; tuberculosis had an RR of 2·30 (2·14–2·48) and EMR of 0·07; and acute hepatitis had an RR of 1·41 (1·23–1·61) and EMR of 0·01. We found a high risk for lower respiratory infections and diarrhoeal diseases in patients with severe mental illness, with RRs of 1·5 (1·46–1·54) and 1·41 (1·27–1·58) and EMRs of 0·28 and 0·02, respectively. Lower respiratory tract diseases had the highest EMR (0·54) in those aged 60 years and above (Figure 1, Figure 2).

**Figure 3:**
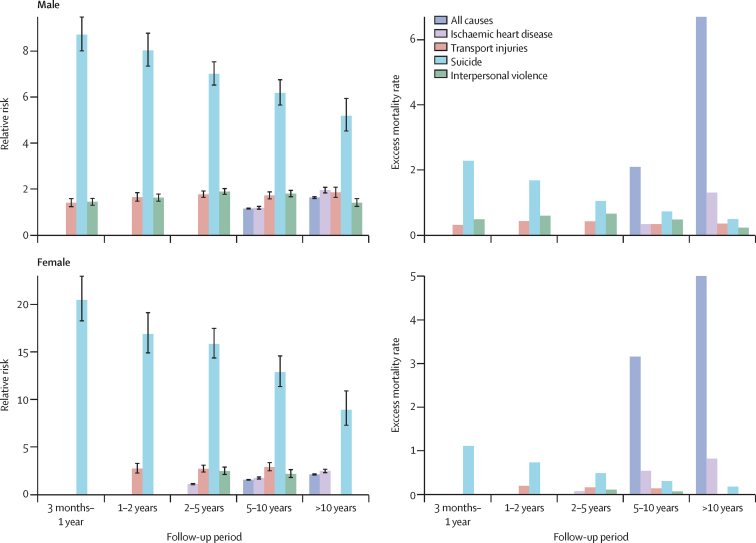
Mortality trends by sex across severe mental illness exposure duration

In the cohort with severe mental illness, NCDs accounted for most of the known-cause deaths (66·5% of all deaths). The RRs from all non-communicable causes of death were high in the total cohort with severe mental illness and in each demographic subgroup (Figure 2, Figure 3).

Among non-communicable causes of death in people with severe mental illness, cardiovascular disease had the highest EMR, accounting for 41·4% of all NCD deaths. In particular, ischaemic heart disease had an RR of 1·39 (95% CI 1·36–1·42) and an EMR of 0·41. The RR for ischaemic heart disease was high in each demographic group and was higher for females than for males; the risk decreased with age, with the highest RR in the younger age.

Among neurological disorders, idiopathic epilepsy had the highest RR (5·7, 95% CI 5·28–6·15), followed by Parkinson's disease (1·68, 1·44–1·97) and Alzheimer's disease and other dementias (1·18, 1·09–1·28; Figure 1, Figure 2).

Among other specific non-communicable causes of death, people with severe mental illness had an RR of 7·61 (95% CI 6·23–9·28) for drug use disorders, 2·93 (2·76–3·12) for alcohol use disorders, 1·84 (1·67–2·03) for pancreatitis, and 1·52 (1·45–1·58) for cirrhosis and other chronic liver diseases. The highest EMRs were found for cirrhosis and other chronic liver diseases (0·14) and alcohol use disorders (0·13). Diabetes had an RR of 1·08 (95% CI 1·05–1·12). The diabetes RR was significant among females and younger adults (Figure 1, Figure 2).

**Figure 4:**
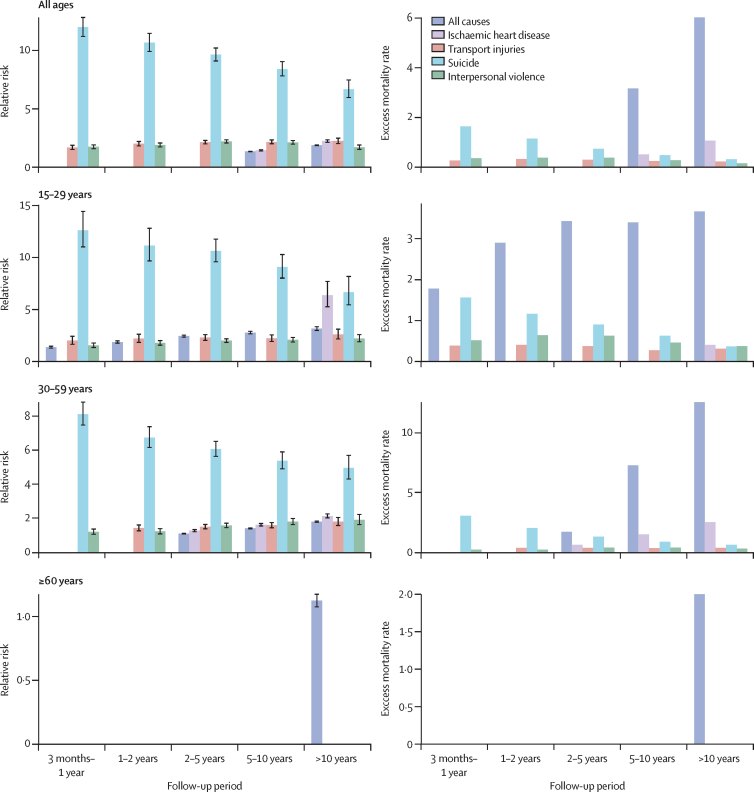
Mortality trends by age across severe mental illness exposure duration

We selected four causes of death (ischaemic heart disease, transport injuries, suicide, and interpersonal violence) and the total from all causes of death in people with severe mental illness to assess their changes over time (Figure 3, Figure 4). The RR and EMR for suicide in people with severe mental illness decreased over the follow-up period and were highest in the first year after the initial diagnosis in all ages and sexes. In particular, the RR decreased from 12·00 (95% CI 11·21–12·84) in between 3 months and 1 year after diagnosis to 6·68 (95% CI 5·96–7·47) 10 years after diagnosis. Across different age groups, the highest EMR of all causes of death was for those 15–29 years over the different periods of follow-up. In those aged 30–59 years, the EMR of all causes of death increased with follow-up, especially after 5–10 years. Interpersonal violence had a high RR in all age groups, with the highest EMR in those aged 15–29 years. Ischaemic heart disease affected mostly those aged 30–59 years, especially after 2 years of follow-up after admission, but the RR for ischaemic heart disease was high in those aged 15–29 years.

## Discussion

In this study, we found that patients receiving inpatient treatment for severe mental illness in the SUS in Brazil had an increased risk of mortality compared with people hospitalised for other clinical conditions. The RR we observed (1·27, 95% CI 1·27–1·28) was lower than the pooled RR of 2·22 found in a meta-analysis of people with mental disorders.^6^ However, our control group was hospital inpatients, so RRs could be substantially underestimated compared with the general population. Our findings show that in Brazil, a middle-income country, the pattern of mortality risk from some conditions differed from that in HICs.^6^, ^7^, ^19^ In this regard, our findings indicated an excess mortality risk that was systematically and significantly high for injuries (eg, interpersonal violence and road injuries), infections (eg, tuberculosis, acute hepatitis, and HIV), and chronic disease (eg, epilepsy and ischaemic heart disease). Additionally, we did not find an excess mortality risk associated with cancer in inpatients with severe mental illness. Individuals younger than 59 years were at a higher risk of mortality, especially those younger than 29 years. In the population with severe mental illness, we observed a higher number of deaths among males than among females. However, when we evaluated all mortality causes, females had a greater RR of mortality than did males. We observed higher RRs for females for the majority of diseases, including HIV, cardiovascular disease, ischaemic heart disease, lower respiratory infections, and diabetes, and most injuries, including suicide, fires, drowning, and homicide.

The gap between the mortality of patients with severe mental illness and the general population is frequently discussed in the literature. However, in our study, some points merit attention. We found a high mortality risk for injury, besides suicide, in the population with severe mental illness, especially unintentional causes (fires, road injuries and other transport injuries, drowning, foreign body, and falls). In the intentional causes of injury, besides suicide, we found that patients with severe mental illness, especially females, are highly exposed to interpersonal violence and have an overall RR that is 2·4 times the rate for hospitalised patients without severe mental illness. To our knowledge, no previous study has found such a high risk of homicide as our study.^24^ These findings highlight the vulnerability of people with severe mental illness in Brazil, particularly in relation to being exposed to violence, especially females. A high lifetime prevalence of sexual violence against patients with mental illness in Brazil was observed (19·8%), which was higher among females (26·6%) than among males (12·5%).^25^ Brazil has a high rate of violent death versus other causes of death.^11^ This reality of higher risk and excess mortality from injury, particularly homicide, indicates differences from studies in HICs and reveals the social and economic gaps that Brazil, and probably other LMICs, must face.^26^

In accordance with other studies, we found that suicide was the most frequent cause of unnatural death, followed by accidents and then homicide.^19^ It is important to consider that some unnatural deaths of unintentional or indeterminate intent might be misclassified as suicide deaths.^27^ In our study, we redistributed the unknown causes of death using the GBD methodology to redistribute garbage codes, reducing the misclassified suicide deaths. We found that the RR trend in mortality from suicide was higher after hospitalisation and decreased with years of follow-up, but remained higher compared with others cause of death.

For communicable diseases, the RR of death from tuberculosis was very high, indicating the poor health conditions in the population with severe mental illness in Brazil. Many previous studies have not addressed this association between severe mental illness and poor health conditions. In LMICs, the rates of common mental disorders are highest among those with tuberculosis or HIV, and are associated with higher morbidity and mortality, increased community transmission, and poor adherence to antiretroviral therapies.^9^ We found that for HIV the overall RR for mortality was 2·03 for people with severe mental illness, and was higher in females. Adults with mental illness in Brazil often face challenges such as homelessness, a history of sexual violence, and increased risk of sexually transmitted diseases.^28^ We found a high risk of mortality for lower respiratory infections, diarrhoeal diseases, and acute hepatitis, which indicated a high risk of death from preventable infection in people with severe mental illness in Brazil. Meta-regression analyses suggested that more recently published studies report lower rates of accidental, natural, and infectious disease mortality in patients with mental ill health.^19^ All these causes of death are preventable, and deaths from these conditions indicate that this population did not receive the necessary health care. Many patients with severe mental illness do not have regular access to primary care and many mental health-related stigmas exist in health care, which can impact the quality of care these patients receive. Patients with mental ill health have reported feeling dismissed or demeaned, and being excluded from the decision making process while receiving care.^29^

For non-communicable diseases, we noted the high risk of mortality from cardiovascular disease, especially ischaemic heart disease. We observed an EMR of 1·38 in those aged 30–59 years, which indicates ischemic heart disease is the cause of death with the highest excess mortality for people with severe mental illness, after suicide. Moreover, the excess ischaemic heart disease mortality was notably high among female patients. Some researchers suggest an explanation for this excess ischaemic heart disease mortality is that patients with severe mental illness might have lower quality of and poorer access to treatment for cardiovascular diseases.^2^, ^26^, ^30^, ^31^

People with severe mental illness have higher cardiovascular disease risk factors, such as high smoking prevalence, sedentary lifestyle, and obesity, as well as metabolic side-effects of antipsychotic treatment.^6^, ^7^, ^19^ Current smoking prevalence among patients with mental ill health in 2007 in Brazil was 53%, much higher than the rate among the general population, which was 22% in 2008.^32^ Many researchers have discussed that people with severe mental illness might have difficulty in reducing or removing modifiable risk factors because of a lack of motivation, poor communication skills, and poor medication compliance, making it challenging to achieve better outcomes.^6^, ^7^, ^19^ Additionally, Charlson and colleagues^33^ found that the pooled RR of developing ischaemic heart disease in those with major depression was 1·56 (95% CI 1·30–1·87). This finding reaffirms the need to assess for depression in patients with ischaemic heart disease, or those who are at a high risk of developing it.^33^

We also found a higher risk of mortality from diabetes among females and younger adults with severe mental illness. Some previous research found an increased risk of diabetes mortality in patients with schizophrenia in the USA.^24^ A higher prevalence of diabetes and abnormal glucose metabolism have been found in inpatients with mental illness compared with the general population and this might be linked to antipsychotic medication use in these patients.^34^

Unlike patterns in HICs, we did not find an increase in the risk of cancers. People with mental disorders have inequitable access to health care, with reduced early cancer detection and a reduced likelihood of receiving screening for breast, cervical, and colorectal cancer compared with the general population.^30^, ^35^ A possible explanation is that patients with severe mental illness in Brazil die from other causes, such as cardiovascular disease, before the expected age of death from cancer.

In our study, neurological conditions, such as idiopathic epilepsy, showed a high RR in people with severe mental illness, especially in younger people, different from the pattern in HICs. Excess mortality in people with mental disorders is reported to be up to six times higher in LMICs,^36^ particularly in poorer, rural populations.^37^

We found a high risk of death from drug and alcohol use disorders in our study in accordance with previous studies.^26^, ^38^ Substance use disorders in patients with schizophrenia have been associated with violent behaviour toward others, suicide, and increased risk of contact with the legal system.^39^, ^40^ Overall, substance misuse comorbidity substantially complicates the course and management of patients with severe mental illness. In Brazil, a study found higher rates of both past-year (11·4%) and lifetime (25·4%) illicit drug use among patients with mental ill health.^41^

The EMR was excessively high in young people in our study supporting previous findings.^25^ The persistent gap in life expectancy for those with severe mental illness should be viewed as an example of discrimination, and points to a need for increased equity among those living with mental illness compared with the general population.^42^

People with severe mental illness are exposed to worse cultural, sociological, and economic factors, such as less healthy lifestyles, poorer socioeconomic circumstances, and the social consequences of mental illness.^25^, ^28^, ^41^, ^43^ Additionally mortality in people with mental disorders is likely to be related to various causes, including behavioural factors and patterns in modifiable risk factors, access to and quality of health-care systems, and social determinants of health, such as poverty and lack of social support.^6^, ^7^

This study has some limitations. The use of inpatients without severe mental illness as a control group underestimates the RRs when compared with the general population. Additionally, EMR is underestimated due to right-point imputation; in other words, we assume right-censored individuals live forever. Although we do not believe censoring effects significantly affected our results, future analyses should conduct a survival analysis to avoid censoring biases. Our study evaluated patients with mental ill health who were admitted to psychiatric hospitals and did not assess data from outpatients. Data from a multicentre study in Brazil^43^ indicate that, among 1577 patients who were being treated in a Public Mental Health Outpatient Clinic, 50% had a history of previous admissions to psychiatric hospitals in their lifetime. The database used presented some variations in the quality and coverage in different geographical areas and we did not have access to data after 2015. The database did not include the private health system. Coverage within the public health system for many long-term illnesses, including severe mental illness, is probably higher than 70%; however, data do not yet exist to indicate an accurate percentage. Lastly, we have no means of validating the accuracy of severe mental illness diagnosis and some misclassification in health records using ICD codes might have occurred.

In conclusion, this study shows that inpatients with severe mental disorders in Brazil have a higher risk of death compared with inpatients with other diagnoses, particularly from ischaemic heart disease. Beyond suicide prevention, interventions for many deaths caused by accidents, injuries, or homicides are urgently required, notably interpersonal violence. Young patients with mental disorders should be a priority group, considering that most causes of death observed in this age group in this study are preventable. Each psychiatric admission presents an opportunity for general medical assessment, enabling preventive health measures that can save lives. The results of this study indicate the need to remove barriers to the integration of medical and mental health care for patients with severe mental illness to reduce excess mortality.

## Data sharing

The findings of this study are supported by data that are not publicly available due to restrictions from the data provider. Non-publicly available data were used under license for the current study but might be available from the authors upon reasonable request and with the permission of the data provider.

## Declaration of interests

All other authors declare no competing interests.
