# Supplementary material for: All-cause and cause-specific mortality among people with severe mental illness in Brazil's public health system, 2000–15: a retrospective study
Source: Lancet Psychiatry. 2022 Oct;9(10):771–81. doi: 10.1016/S2215-0366(22)00237-1 (PMC9477749; doi:10.1016/S2215-0366(22)00237-1)
Supplement: Supplementary appendix [file mmc1.pdf]

THE LANCET
Psychiatry

## Supplementary appendix

This appendix formed part of the original submission and has been peer reviewed. We post it as supplied by the authors.

Supplement to: Melo APS, Dippenaar IN, Johnson SC, et al. All-cause and cause-specific mortality among people with severe mental illness in Brazil's public health system, 2000–15: a retrospective study. *Lancet Psychiatry* 2022; published online Aug 11.
https://doi.org/10.1016/S2215-0366(22)00237-1
.

# Supplementary Appendix: All-cause and cause-specific mortality among people with severe mental illness in Brazil, 2000-2015

## Table of Contents

Table of Contents ........ 1
Detailed Methods ........ 3
Construction of the Linkage Database ........ 3
Data cleaning ........ 4
Cause mapping and redistribution ........ 4
Follow-up period definition ........ 5
Relative risk and excess mortality calculation ........ 5
References ........ 7
Supplemental Tables ........ 8
Appendix table 1: Summary of mortality cause map. International Classification of Diseases (ICD-10) Codes provided for intermediate or non-fatal diagnoses. ........ 8
Supplemental Figures ........ 41
Results for Depressive Disorders, Bipolar Disorder, and Schizophrenia ........ 41
Appendix figure 1: Significant relative risk of mortality and excess mortality rate for all ages by sex with depressive disorder exposure longer than three months. Causes of death with fewer than 100 cases in the given age and sex are excluded. ........ 41
Appendix figure 2: Significant relative risk of mortality and excess mortality rate for all sexes by age with depressive disorder exposure longer than three months. Causes of death with fewer than 100 cases in the given age and sex are excluded. ........ 42
Appendix figure 3: Significant relative risk of mortality and excess mortality rate for all ages by sex with bipolar disorder exposure longer than three months. Causes of death with fewer than 100 cases in the given age and sex are excluded. ........ 43

Appendix figure 4: Significant relative risk of mortality and excess mortality rate for all sexes by age with bipolar disorder exposure longer than three months. Causes of death with fewer than 100 cases in the given age and sex are excluded. ........ 44

Appendix figure 5: Significant relative risk of mortality and excess mortality rate for all ages by sex with schizophrenia exposure longer than three months. Causes of death with fewer than 100 cases in the given age and sex are excluded. ........ 45

Appendix figure 6: Significant relative risk of mortality and excess mortality rate for all sexes by age with schizophrenia exposure longer than three months. Causes of death with fewer than 100 cases in the given age and sex are excluded. ........ 46

GATHER Compliance: Guidelines for Accurate and Transparent Health Estimates Reporting........ 47

# Detailed Methods

## Construction of the Linkage Database

A record linkage model was used for the main SUS information systems: (a) the Hospital Information System (SIH) and (b) the Mortality Information System (SIM) with a technique developed by Fellegi & Sunter,[1] which includes a deterministic and probabilistic deduplication of records used to build The Brazilian National Database of Health (BNDH) centered on the individual.[2] The BNDH was built following these steps: pre-processing; deterministic de-duplication intra-system; deterministic de-duplication inter-system; probabilistic deduplication; linkage quality analysis and classification; and clustering. The fields used in the pairing were (1) Social Security, (2) full name, (3) sex, (4) date of birth, (5) code of the Brazilian Institute of Geography and Statistics (IBGE) of the municipality of residence, and (6) street address, city of residence. The methods and results of parameter setting needed to execute the probabilistic deduplication of the databases to create a BNDH were described in detail elsewhere.[2] Here we summarise the main procedures. We performed cleaning and standardisation to ensure data quality for the deduplication process, we standardised the attributes like excluding invalid names, dates and incorrect social security numbers. After processing the deterministic deduplication intrasystem for each database, we gathered all data into the table of personal identification for the period of 2000-2015. We used these records to process the deterministic deduplication inter-system. The probabilistic deduplication process started with an estimation of the probabilities. We randomly sampled 1,000,000 records and carried out a probabilistic deduplication with parameters used by De Queiroz et al.[3] In this method, the values are distributed in two main variables: m and u. The value m is the value measured for an attribute comparison when the pair is considered true and the attribute agrees on that pair. The value u is the value measured for an attribute comparison when the pair is considered false yet the attribute still does agree on this pair. At this step, we recalculated the values of “m”, “u” and “missing” for this new dataset. We executed this process repeatedly and by the third round, it was possible to see a trend towards stabilisation of the probabilities. Values of m and u, and respective weights of agreement and disagreement, for the probabilistic deduplication of records were calculated for each one of the following attributes: patient’s name, mother’s name, father’s name, sex, social security number, date of birth, state, city, and zip code. We established nine blocking strategies aiming to cover the whole dataset possible comparisons. In the linkage quality analysis, we separated the amplitudes of the scores into 5-point ranges, resulting in 28 random samples with 432 pairs each. Thus, the first range has scores between 135-140, and the last range between -5 and 0. In a clerical review, those pairs were manually inspected and classified as correct or incorrect by two independent reviewers and kappa statistics were used to guarantee the quality of the linkage. The review showed a mean agreement between the ranges of 97.4% of cases (Kappa = 0.952). Considering the reviewers’ decision, the frequency of true pairs for each range evaluated was calculated. A specific range was defined as a cut-off point, and a score of 30, on the lower side, was selected. By taking this score, the estimated error was calculated: for false positive error, the estimated value was 3.3%, while the false negative error was estimated at 12.3%. To preserve the confidentiality of clinical events, medical treatments performed, and causes of death, databases were separated into distinct tables – one with personal identification data and the other with all health events and causes of death. During the linkage, the researchers only had access to the table with personal data. To preserve patient privacy, the in-depth search graph algorithm was used, which generates unique and anonymous identifiers. After that, these unique and anonymous identifiers were linked with health events and causes of death. The National Database of Health centered on the individual, a Brazilian cohort database (2000–2015) spanning 15 years of historical data, consists of 1.3 billion records in

this period, covering a population of approximately 200 million inhabitants.[2] More details on record linkage process can be found in the publication from Guerra Junior at al.[2]

## Data cleaning

To counteract the known false positive linkage rate from the probabilistic linkage process,[2] we excluded some patient records from analysis. Any linked patients with ambiguous birthdates (unknown birthdate or with multiple birth years assigned to the same patient), ambiguous death dates (unknown death date or multiple death dates), multiple death certificates, or a record of death in SIH-SUS without an accompanying death record in SIM were excluded from further analysis. A total of 4.1% (3,593,863) of the linked patients were excluded in this way. To limit our study to just hospital inpatients and avoid inflating our study with otherwise healthy individuals, we also excluded obstetrics-related admissions. Specifically, we excluded any admission recorded in SIH-SUS with ICD-10 codes O80-O80.9, O84, O84.0, O84.8, and O84.9 listed in either the primary or secondary diagnosis. A total of 12.3% (18,590,310) of admissions were excluded this way. Finally, we excluded one patient with unknown age and 357 patients without a recorded sex. In total, we analysed records from 72,021,918 patients as a subset from the 87,561,735 patients in the raw data.

Since we are interested in the mortality outcomes following the onset of mental disorders, we only considered the earliest observed diagnosis of a specific cause for each individual rather than repeated diagnoses with the same cause. In other words, we excluded any diagnosis for a patient if that patient were admitted with the same cause earlier in our observation window. Data processing was performed using the Dask library.[4]

## Cause mapping and redistribution

We mapped ICD-10 codes to a cause list for easier analysis. We classified some codes as “garbage codes” which are either not specific enough, an immediate or intermediate cause of death, or an impossible cause for death. Approximately 23.5% of all deaths were assigned to garbage codes (table 1). Following GBD methodology, we performed garbage code redistribution for any garbage-coded deaths.[5] If a garbage code was assigned as an underlying cause, then fractions of this death would be assigned to each of the target causes in the redistribution process since each death in the GBD could be allocated to only one underlying cause as per ICD categorisation of causes of death. The only mental disorders identified as underlying causes of death were anorexia nervosa and bulimia nervosa. The underlying causes of death cannot be due to mental illness per se, rather it is the consequence of the simultaneous presence of comorbid physical health problems, such as cardiovascular, respiratory, metabolic, infectious diseases and cancer Fiorillo, A., & Sartorius, N. (2021).[6] See appendix table 1 for a full list of underlying causes of death analysed as well as the set of ICD-10 codes labelled as garbage codes.

Since some ICD codes cannot be an underlying cause of death but can be a possible intermediate or non-fatal diagnosis, we used different maps for mortality and morbidity. For example, “Major depressive disorder, single episode (F32)” is an ill-defined cause of death but a possible

intermediate cause or a non-fatal diagnosis. We considered intermediate and immediate causes on the death certificate and all hospital diagnoses equivalently.

## Follow-up period definition

To compare patients with SMI against a population more representative of the overall population, we excluded any diagnosis that was followed by death within three months. Then, for each patient and non-fatal diagnosis, we analysed the mortality outcomes at any point during the remaining observation period after three months and used a cutoff point of at least 100 cases from each cause of death. Thus, our follow-up period was three months up to 15 years and two months for all-cause and cause-specific mortality.

To analyse the trend of mortality over time, we selected five additional follow-up periods to examine: 3 months–1 year, 1–2 years, 2–5 years, 5–10 years, and >10 years (main text figures 3 and 4). For each follow-up period we only considered mortality events observed within the given time frame after a diagnosis. We considered a lack of an observed death within the time frame to indicate survival. Each follow-up period was otherwise treated identically in the analysis.

## Relative risk and excess mortality calculation

To quantify both the relative and absolute increase in risk imposed by SMI, we used two measures to quantify excess mortality: relative risk (RR) and excess mortality rate (EMR).7 In the current study, the relative risk is the ratio of two mortality rates where the numerator is the mortality rate in the population with SMI and the denominator is the mortality rate in the population without SMI. In contrast, the EMR is the difference between the mortality rate in the general population and the mortality rate in the unexposed population. It has also been named the "population attributable risk", though exact terminology varies widely. As an example, an EMR of 4 per 100 000 would indicate the mortality rate increased by 4 because of the increased mortality burden of SMI. It is important to note that the RR measures the relative increase in mortality, but the EMR measures the absolute increase in mortality, thus giving two complementary perspectives on the burden of SMI.

We constructed a 2x2 confusion matrix for each combination of age, sex, underlying cause of death, and nonfatal diagnosis (SMI, bipolar disorder, depression, and schizophrenia) as follows:

**Confusion matrix for RR and EMR calculation:**

| | | **Died of a given cause** | | **Totals** |
|---|---|---|---|---|
| | | **Yes** | **No** | |
| **Diagnosed with a given cause** | **Yes** | $a$ | $b$ | $n_1 = a + b$ |
| | **No** | $c$ | $d$ | $n_2 = c + d$ |
| | **Totals** | $m_1 = a + c$ | $m_2 = b + d$ | $N = n_1 + n_2$ |

Each cell in the above table represents the number of patients with the condition. For example, when the diagnosis under consideration is any SMI and the cause of death under consideration is suicide, then $b$ is the number of patients who were diagnosed with SMI and either did not die from suicide or did not die at all during our observation period. The total population $N$ is the number of patients recorded with the given age and sex. We calculated RR as

$$RR = \frac{\frac{a}{n_1}}{\frac{c}{n_2}}$$

with

$$\mathrm{Var}(RR) = \frac{1}{a} - \frac{1}{n_1} + \frac{1}{c} - \frac{1}{n_2}$$

We calculated EMR as

$$EMR = \frac{m_1}{N} - \frac{c}{n_2}$$

We performed subgroup analyses on age, sex, and underlying cause. We grouped patients by age group (15-29, 30-59, and 60+ years of age) and sex (male and female) and then calculated RR and EMR for every age/sex combination and for each underlying cause of death. See appendix table 1 for the causes we considered in our analyses. Each age, sex, and underlying cause of death combination was treated identically throughout the analysis to maximize comparability between groups. We report only on the most detailed causes listed in the hierarchy.

We ignored censoring effects since we are only interested in the relative difference in survival between patients with and without SMI. Since the censoring time is not correlated with an SMI diagnosis, any underestimation of the mortality risk from right censoring would be similar in both the diagnosed and undiagnosed groups. Therefore, we do not expect the relative risk to suffer from censoring effects, although EMR would be slightly underestimated.

## References

1 Fellegi IP, Sunter AB. A Theory for Record Linkage. *J Am Stat Assoc* 1969; **64**: 1183–210.

2 Guerra Junior AA, Pereira RG, Gurgel EI, *et al.* Building the national database of health centred on the individual: administrative and epidemiological record linkage - brazil, 2000-2015. *Int J Popul Data Sci*; **3**: 446.

3 De Queiroz OV, Guerra Júnior AA, Machado CJ, Andrade EIG, Meira Junior W; Acúrcio FA, Santos Filho W, Cherchiglia ML. Record linkage of large data sources: parameter estimation and results validation, applied to the linkage of high complexity procedures authorizations with the hospital information system. *Cad Saúde Coletiva UFRJ* 2010; **18**: 298–308.

4 Dask: Scalable analytics in Python. https://dask.org/ (accessed May 13, 2022).

5 Johnson SC, Cunningham M, Dippenaar IN, *et al.* Public health utility of cause of death data: applying empirical algorithms to improve data quality. *BMC Med Inform Decis Mak* 2021; **21**: 175.

6 Fiorillo A, Sartorius N. Mortality gap and physical comorbidity of people with severe mental disorders: the public health scandal. *Ann Gen Psychiatry* 2021; **20**: 52.

## Supplemental Tables

Appendix table 1: Summary of mortality cause map. International Classification of Diseases (ICD-10) Codes provided for intermediate or non-fatal diagnoses.

| Level 1 | Level 2 | Level 3 | Level 4 | ICD-10 Codes |
|---|---|---|---|---|
| HIV/AIDS and sexually transmitted infections | | | | A57-A58, A60-A60.9, A63-A63.8, B20-B24.9, B63, D80-D84.9, D89.8-D89.9, F02.4, I98.0, K67.0-K67.2, M03.1, M73.0-M73.1, O98.7 |
| | HIV/AIDS | | | B20-B24.9, D80-D84.9, D89.8-D89.9, F02.4, O98.7 |
| | Sexually transmitted infections excluding HIV | | | A57-A58, A60-A60.9, A63-A63.8, B63, I98.0, K67.0-K67.2, M03.1, M73.0-M73.1 |
| Respiratory infections and tuberculosis | | | | A10-A19.9, A48.1, A70, B34.2, B90-B90.9, B96.0-B96.1, B97.2-B97.6, H70-H70.9, J00-J06.9, J09-J22.9, J36-J36.0, J91.0, K67.3, K93.0, M49.0, N74.1, P23-P23.9, P37.0, U04.9, U07-U07.2, U84.3 |
| | Tuberculosis | | | A10-A19.9, B90-B90.9, K67.3, K93.0, M49.0, N74.1, P37.0, U84.3 |
| | Lower respiratory infections | | | A48.1, A70, B34.2, B96.0-B96.1, B97.2-B97.6, J09-J22.9, J91.0, P23-P23.9, U04.9, U07-U07.2 |
| | Upper respiratory infections | | | J00-J06.9, J36-J36.0 |

| | | | | |
|---|---|---|---|---|
| | Otitis media | | | H70-H70.9 |
| Enteric infections | | | | A00-A09.9, K52.1-K52.3, R19.7 |
| | Diarrheal diseases | | | A00-A00.9, A02-A02.0, A02.8-A07, A07.2-A07.4, A08-A09.9, K52.1-K52.3, R19.7 |
| | Typhoid and paratyphoid | | | A01-A01.4 |
| | Invasive Non-typhoidal Salmonella (iNTS) | | | A02.1-A02.2 |
| | Other intestinal infectious diseases | | | A07.0-A07.1, A07.8-A07.9 |
| Neglected tropical diseases and malaria | | | | A68-A68.9, A69.2-A69.9, A75-A75.9, A77-A79.9, A82-A82.9, A90-A98.8, B33.0-B33.1, B50-B57.5, B60-B60.8, B65-B67.9, B69-B72.0, B74.3-B83.9, B89, K93.1, P37.1, P37.3-P37.4, U06-U06.9 |
| | Malaria | | | B50-B54.0, P37.3-P37.4 |
| | Chagas disease | | | B57-B57.5, K93.1 |
| | Leishmaniasis | | | B55-B55.9 |
| | Schistosomiasis | | | B65-B65.9 |
| | Cysticercosis | | | B69-B69.9 |

| | | | | |
|---|---|---|---|---|
| | Cystic echinococcosis | | | B67-B67.4, B67.8-B67.9 |
| | Dengue | | | A90-A91.9 |
| | Yellow fever | | | A95-A95.9 |
| | Intestinal nematode infections | | | B76-B82.9, B83.9 |
| | Food-borne trematodiases | | | B66-B66.9, B72.0 |
| | Other neglected tropical diseases | | | A68-A68.9, A69.2-A69.9, A75-A75.9, A77-A79.9, A82-A82.9, A92-A94.0, A96-A98.8, B33.0-B33.1, B56-B56.9, B60-B60.8, B67.5-B67.7, B70-B72, B74.3-B75, B83-B83.8, B89, P37.1, U06-U06.9 |
| Other infectious diseases | | | | A20-A39.9, A42-A45.9, A47-A48, A48.2, A48.4-A49, A49.2-A56.8, A59, A65-A65.0, A67.7, A69-A69.1, A74, A74.8-A74.9, A80-A81.9, A83-A89.9, B00-B06.9, B10-B10.8, B15-B16.2, B17-B19.9, B25-B27.9, B29.4, B33, B33.3-B33.8, B37-B49.9, B58-B59.9, B61-B62, B64, B68-B68.9, B91, B94.1, B95-B95.5, B96, B96.2-B97.1, B97.7-B99.9, D70.3, D89.3, F02.1, F07.1, G00.0-G00.8, G03-G03.8, G04-G09.9, G14-G14.6, I00, I02, I02.9, I98.1, K67.8, K75.3, K76.3, K77.0, M49.1, M89.6, P35-P35.9, P37, P37.2, P37.5-P37.9, U82-U84, U85-U89, Z16-Z16.3 |
| | Meningitis | | | A39-A39.9, A87-A87.9, G00.0-G00.8, G03-G03.8, G06-G09.9 |
| | Encephalitis | | | A83-A86.4, B94.1, F07.1, G04-G05.8 |
| | Diphtheria | | | A36-A36.9 |

| | | | | |
|---|---|---|---|---|
| | Pertussis | | | A37-A37.9 |
| | Tetanus | | | A33-A35.0 |
| | Measles | | | B05-B05.9 |
| | Varicella and herpes zoster | | | B01-B02.9, P35.8 |
| | Acute hepatitis | | | B15-B16.2, B17-B19.9, P35.3 |
| | Other unspecified infectious diseases | | | A20-A32.9, A38-A38.9, A42-A45.9, A47-A48, A48.2, A48.4-A49, A49.2-A56.8, A59, A65-A65.0, A67.7, A69-A69.1, A74, A74.8-A74.9, A80-A81.9, A88-A89.9, B00-B00.9, B03-B04, B06-B06.9, B10-B10.8, B25-B27.9, B29.4, B33, B33.3-B33.8, B37-B49.9, B58-B59.9, B61-B62, B64, B68-B68.9, B91, B95-B95.5, B96, B96.2-B97.1, B97.7-B99.9, D70.3, D89.3, F02.1, G14-G14.6, I00, I02, I02.9, I98.1, K67.8, K75.3, K76.3, K77.0, M49.1, M89.6, P35-P35.2, P35.9, P37, P37.2, P37.5-P37.9, U82-U84, U85-U89, Z16-Z16.3 |
| | | Rubella | | P35.0 |
| Maternal and neonatal disorders | | | | C22.3-C22.4, N96, N98-N98.9, O00-O79, O81-O83.9, O84.1-O84.2, O85-O98.6, O98.8-P04.2, P04.5-P22.9, P24-P34.2, P36-P36.9, P38-P94.9, P96-P99.9 |
| | Maternal disorders | | | C22.3-C22.4, N96, N98-N98.9, O00-O79, O81-O83.9, O84.1-O84.2, O85-O98.6, O98.8-O99.9 |
| | | Maternal hemorrhage | | O20-O20.9, O43.2, O44-O46.9, O62-O62.9, O67-O67.9, O70, O72-O72.3 |

| | | | | |
|---|---|---|---|---|
| | | Maternal sepsis and other maternal infections | | O23-O23.9, O85-O86.8, O91-O91.2 |
| | | Maternal obstructed labor and uterine rupture | | O32-O33.9, O64-O66.9, O71-O71.9 |
| | | Maternal abortion and miscarriage | | N96, O01-O07.9 |
| | | Ectopic pregnancy | | O00-O00.9 |
| | | Indirect maternal deaths | | O24-O25.3, O98-O98.6, O98.8-O99.9 |
| | | Late maternal deaths | | O96-O97.9 |
| | | Other direct maternal disorders | | N98-N98.9, O08-O19, O21-O22.9, O26-O31.8, O34-O43.1, O43.8-O43.9, O47-O61.9, O63-O63.9, O68-O69.9, O70.0-O70.9, O73-O79, O81-O83.9, O84.1-O84.2, O87-O90.9, O92-O95.9 |
| | Neonatal disorders | | | P00-P04.2, P04.5-P22.9, P24-P34.2, P36-P36.9, P38-P94.9, P96-P99.9 |
| | | Neonatal preterm birth | | P01.0-P01.1, P05-P05.9, P07-P07.3, P22-P22.9, P25-P28.9 |
| | | Neonatal encephalopathy due to birth asphyxia and trauma | | P01.7, P02-P03.9, P10-P15.9, P20-P21.9, P24-P24.9 |

| | | | | |
|---|---|---|---|---|
| | | Neonatal sepsis and other neonatal infections | | P36-P36.9, P38-P39.9 |
| | | Other neonatal disorders | | P00-P01, P01.2-P01.6, P01.8-P01.9, P04-P04.2, P04.5-P04.9, P06, P08-P09, P16-P19.9, P29-P34.2, P40-P94.9, P96-P99.9 |
| Nutritional deficiencies | | | | C83.4-C83.7, D50.1-D50.8, D51-D52.0, D52.8-D53.9, E00-E02, E40-E46.9, E51-E61.9, E63-E64.0, E64.2-E64.9, M12.1 |
| | Protein-energy malnutrition | | | E40-E46.9, E64.0 |
| | Dietary iron deficiency | | | D50.1-D50.8 |
| | Other nutritional deficiencies | | | C83.4-C83.7, D51-D52.0, D52.8-D53.9, E00-E02, E51-E61.9, E63-E64, E64.2-E64.9, M12.1 |
| Non-communicable diseases | | | | A46-A46.0, A66-A67.3, A67.9, B33.2, B86, C00-C13.9, C15-C22.2, C22.5-C22.8, C23-C25.9, C30-C34.9, C37-C41.9, C43-C54.9, C56-C57.8, C58-C58.0, C60-C63.8, C64-C67.9, C68.0-C68.8, C69-C73.9, C75-C75.8, C81-C83.3, C83.8-C86.6, C88-C96.9, D00.1-D00.2, D01.0-D01.3, D02.0-D02.3, D03-D06.9, D07.0-D07.2, D07.4-D07.5, D09.0, D09.2-D09.3, D09.8, D10.0-D10.7, D11-D12.9, D13.0-D13.7, D14.0-D14.3, D15-D16.9, D22-D27.9, D28.0-D28.7, D29.0-D29.8, D30.0-D30.8, D31-D36, D36.1-D36.7, D37.1-D37.5, D38.0-D38.5, D39.1-D39.2, D39.8, D40.0-D40.8, D41.0-D41.8, D42-D43.9, D44.0-D44.8, D45-D47.9, D48.0-D48.6, D49.2-D49.4, D49.6, D55-D58.9, D59.1, D59.3-D59.5, D60-D61.9, D63.1, D64.0, D66-D67, D68.0-D69.4, D69.6-D69.8, D70-D70.0, D70.4-D75.8, D76-D77, D86-D86.9, D89-D89.2, E03-E03.1, E03.3-E06.3, |

| | | | | |
|---|---|---|---|---|
| | | | | E06.5-E07.1, E08-E08.9, E10-E14.9, E16.1-E16.9, E20-E23.0, E23.2-E24.1, E24.3-E27.2, E27.4-E34, E34.1-E34.8, E65-E66.0, E66.2-E68, E70-E85.2, E88-E88.2, E88.4-E88.9, F00-F02.0, F02.2-F02.3, F02.8-F03.9, F10-F16.9, F18-F18.9, F24, F50.0-F50.5, G10-G13.8, G20-G20.9, G23-G24, G24.1-G25.0, G25.2-G25.3, G25.5, G25.8-G26.0, G30-G31.9, G35-G37.9, G40-G41.9, G45-G46.8, G47.3, G61-G61.9, G62.1, G70-G72, G72.1-G73.7, G90-G90.9, G95-G95.9, H05.0-H05.1, I00.0-I01.9, I02.0, I03-I09.9, I11-I13.9, I16-I25.9, I27.0-I27.2, I28-I28.9, I30-I31.1, I31.8-I43.9, I47-I48.9, I51-I67.3, I67.5-I69.9, I70.2-I70.8, I71-I73.9, I77-I94, I96-I96.9, I98, I98.2-I98.8, I99-I99.9, J23-J35.9, J37-J68.9, J70, J70.8-J79, J81.9-J84.9, J85.9, J87-J89, J90.9-J91, J91.8-J92.9, J93.6, J97-J98.0, J98.4-J99.8, K20-K20.9, K22-K22.6, K22.8-K29.9, K31-K42.9, K44-K52, K52.8-K62.6, K62.8-K64.9, K66.8, K67, K68, K69-K70.3, K71.7, K73-K75.2, K75.4-K76.2, K76.4-K77, K77.8-K90.9, K92, K92.8-K93, K93.8, K96-L05.9, L08-L08.9, L10-L14.0, L51-L51.9, L88-L89.9, L93-L93.2, L97-L98.4, M00-M03.0, M03.2-M03.6, M05-M09.8, M30-M36.8, M40-M43.1, M65-M65.0, M71.0-M71.1, M72.5-M72.6, M80-M82.8, M86.3-M86.4, M87-M87.0, M88-M89.0, M89.5, M89.7-M89.9, N00-N13.9, N15-N16.8, N18-N18.9, N20-N30.3, N30.8-N32.0, N32.3-N32.4, N34-N34.3, N36-N36.9, N39-N39.2, N40-N41.9, N44-N44.0, N45-N45.9, N49-N49.9, N60-N60.9, N72-N72.0, N75-N77.8, N80-N81.9, N83-N83.9, N84.0-N84.1, N87-N87.9, P04.3-P04.4, Q00-Q99.9, R78.0-R78.5, R95-R95.9, X40-X46.9, X49-X49.9, X65-X65.9, Y10-Y19.9 |
| | Neoplasms | | | C00-C13.9, C15-C22.2, C22.5-C22.8, C23-C25.9, C30-C34.9, C37-C41.9, C43-C54.9, C56-C57.8, C58-C58.0, C60-C63.8, C64-C67.9, C68.0-C68.8, C69-C73.9, C75-C75.8, C81-C83.3, C83.8-C86.6, C88-C96.9, D00.1-D00.2, D01.0-D01.3, D02.0- |

| | | | | |
|---|---|---|---|---|
| | | | | D02.3, D03-D06.9, D07.0-D07.2, D07.4-D07.5, D09.0, D09.2-D09.3, D09.8, D10.0-D10.7, D11-D12.9, D13.0-D13.7, D14.0-D14.3, D15-D16.9, D22-D24.9, D26.0-D27.9, D28.0-D28.1, D28.7, D29.0-D29.8, D30.0-D30.8, D31-D36, D36.1-D36.7, D37.1-D37.5, D38.0-D38.5, D39.1-D39.2, D39.8, D40.0-D40.8, D41.0-D41.8, D42-D43.9, D44.0-D44.8, D45-D47.9, D48.0-D48.6, D49.2-D49.4, D49.6, K62.0-K62.1, K63.5, N60-N60.9, N84.0-N84.1, N87-N87.9 |
| | | Lip and oral cavity cancer | | C00-C08.9, C62-C62.1, C71.3-C72, C72.2, D10.0-D10.5, D11-D11.9 |
| | | Nasopharynx cancer | | C11-C11.9, C72.0, D10.6 |
| | | Other pharynx cancer | | C09-C10.9, C12-C13.9, C72.1, D10.7 |
| | | Esophageal cancer | | C15-C15.9, C62.9, C72.8, D00.1, D13.0 |
| | | Stomach cancer | | C16-C16.9, C72.9, D00.2, D13.1, D37.1 |
| | | Colon and rectum cancer | | C18-C21.9, C63-C63.0, C72.3-C72.5, D01.0-D01.3, D12-D12.9, D37.3-D37.5 |
| | | Liver cancer | | C22-C22.2, C22.5-C22.8, C73.2, D13.4 |
| | | Gallbladder and biliary tract cancer | | C23-C24.8, C73.3, D13.5 |
| | | Pancreatic cancer | | C24.9-C25.8, C73.4-C73.5, D13.6-D13.7 |
| | | Larynx cancer | | C30.5-C31.2, C73.9, D02.0, D14.1, D38.0 |
| | | Tracheal, bronchus, and lung cancer | | C31.3-C34.3, C63.1, D02.1-D02.3, D14.2-D14.3, D38.1 |

| | | | | |
|---|---|---|---|---|
| | | Malignant skin melanoma | | C63.2-C63.8, C64-C67.3, C83.2, D03-D03.9, D22-D23.9, D48.5 |
| | | Non-melanoma skin cancer | | D04-D04.9, D49.2 |
| | | Soft tissue and other extraosseous sarcomas | | C38.4-C40.1 |
| | | Breast cancer | | C40.3-C41.9, C43-C44.2, C50.6-C50.9, C69.4-C69.8, C83.3, D05-D05.9, D24-D24.9, D48.6, D49.3 |
| | | Cervical cancer | | C44.3, C53-C53.9, C69.9, D06-D06.9, D26.0 |
| | | Uterine cancer | | C54-C54.9, C70, D07.0-D07.2, D26.1-D26.9 |
| | | Ovarian cancer | | C44.4, C56-C56.9, D27-D27.9, D39.1 |
| | | Prostate cancer | | C61-C61.9, C67.4-C67.9, C68.0-C68.8, C70.1, C81, D07.5, D29.1, D40.0 |
| | | Testicular cancer | | C44.7, C81.0, D29.2-D29.8, D40.1-D40.8 |
| | | Kidney cancer | | C44.8-C45.0, C81.1, D30.0-D30.1, D41.0-D41.1 |
| | | Bladder cancer | | C45.6-C46.5, C70.5, D09.0, D30.3, D41.4-D41.8, D49.4 |
| | | Brain and central nervous system cancer | | C46.9-C47.0, C82-C82.6, D32-D33.9, D35.3-D35.4, D42-D43.9, D49.6 |
| | | Eye cancer | | C69-C69.3 |
| | | Neuroblastoma and other peripheral nervous cell tumors | | C34.4-C34.9, C37-C37.2, C46.6 |

| | | | | |
|---|---|---|---|---|
| | | Thyroid cancer | | C71.1-C71.2, D09.3, D09.8, D34-D34.9, D44.0 |
| | | Hodgkin lymphoma | | C47.2-C50.5, C81.8-C81.9 |
| | | Non-Hodgkin lymphoma | | C82.7-C82.8, C83-C83.1, C83.8-C86.6, C96-C96.9 |
| | | Multiple myeloma | | C88-C90.9 |
| | | Leukemia | | C91-C95.9 |
| | | Other malignant neoplasms | | C17-C17.9, C25.9, C30-C30.3, C37.3-C38.3, C40.2, C44.5-C44.6, C45.1-C45.5, C46.7-C46.8, C47.1, C51-C52.9, C57-C57.8, C58-C58.0, C60-C60.9, C70.0, C70.6-C71.0, C73-C73.1, C73.8, C75-C75.8, C81.2-C81.7, C82.9, D07.4, D09.2, D13.2-D13.3, D14.0, D15-D16.9, D28.0-D28.1, D28.7, D29.0, D30.2, D30.4-D30.8, D31-D31.9, D35-D35.2, D35.5-D36, D36.1-D36.7, D37.2, D38.2-D38.5, D39.2, D39.8, D41.2-D41.3, D44.1-D44.8, D48.0-D48.4 |
| | | Other neoplasms | | D45-D47.9, K62.0-K62.1, K63.5, N60-N60.9, N84.0-N84.1, N87-N87.9 |
| | Cardiovascular diseases | | | B33.2, G45-G46.8, I00.0-I01.9, I02.0, I03-I09.9, I11-I11.9, I16-I25.9, I27.0, I27.2, I28-I28.9, I30-I31.1, I31.8-I43.9, I47-I48.9, I51-I67.3, I67.5-I69.9, I70.2-I70.8, I71-I73.9, I77-I83.9, I86-I89.0, I89.9-I94, I96-I96.9, I98, I98.4-I98.8, I99-I99.9, K75.1 |
| | | Rheumatic heart disease | | I01-I01.9, I02.0, I05-I09.9 |
| | | Ischemic heart disease | | I20-I25.9 |

| | | | | |
|---|---|---|---|---|
| | | Stroke | | G45-G46.8, I60-I67.3, I67.5-I69.9 |
| | | Hypertensive heart disease | | I11-I11.9 |
| | | Non-rheumatic valvular heart disease | | I34-I37.8 |
| | | Cardiomyopathy and myocarditis | | B33.2, I40-I43.9, I51.4 |
| | | Pulmonary Arterial Hypertension | | I27.0, I37.9 |
| | | Atrial fibrillation and flutter | | I48-I48.9 |
| | | Aortic aneurysm | | I71-I71.9 |
| | | Lower extremity peripheral arterial disease | | I70.2-I70.8, I73-I73.9 |
| | | Endocarditis | | I33-I33.9, I38-I39.9 |
| | | Other cardiovascular and circulatory diseases | | I00.0, I03-I04, I16-I19, I27.2, I28-I28.9, I30-I31.1, I31.8-I32.8, I47-I47.9, I51-I51.3, I51.5-I59, I72-I72.9, I77-I83.9, I86-I89.0, I89.9-I94, I96-I96.9, I98, I98.4-I98.8, I99-I99.9, K75.1 |
| | Chronic respiratory diseases | | | D86-D86.2, D86.9, G47.3, J23-J35.9, J37-J68.9, J70, J70.8-J79, J81.9-J84.9, J85.9, J87-J89, J90.9-J91, J91.8-J92.9, J93.6, J97-J98.0, J98.4-J99.8 |
| | | Chronic obstructive pulmonary disease | | J41-J44.9 |

| | | | | |
|---|---|---|---|---|
| | | Pneumoconiosis | | J60-J65.0 |
| | | Asthma | | J45-J46.9 |
| | | Interstitial lung disease and pulmonary sarcoidosis | | D86-D86.2, D86.9, J84.9 |
| | | Other chronic respiratory diseases | | G47.3, J23-J35.9, J37-J40.9, J47-J59, J66-J68.9, J70, J70.8-J79, J81.9-J84.8, J85.9, J87-J89, J90.9-J91, J91.8-J92.9, J93.6, J97-J98.0, J98.4-J99.8 |
| | Digestive diseases | | | I84-I85.9, I98.2, K20-K20.9, K22-K22.6, K22.8-K29.9, K31-K42.9, K44-K52, K52.8-K62, K62.2-K62.6, K62.8-K63.4, K63.8-K64.9, K66.8, K67, K68, K69-K70.3, K71.7, K73-K75.0, K75.2, K75.4-K76.2, K76.4-K77, K77.8-K90.9, K92, K92.8-K93, K93.8, K96-K99, M09.1 |
| | | Cirrhosis and other chronic liver diseases | | I85-I85.9, I98.2, K70-K70.3, K71.7, K73-K75, K75.2, K75.4-K76.2, K76.4-K76.9, K77.8 |
| | | Upper digestive system diseases | | K25-K29.9 |
| | | Appendicitis | | K35-K37.9, K38.3-K38.9 |
| | | Paralytic ileus and intestinal obstruction | | K56-K56.9 |
| | | Inguinal, femoral, and abdominal hernia | | K40-K42.9, K44-K46.9 |

| | | | | |
|---|---|---|---|---|
| | | Inflammatory bowel disease | | K50-K52, K52.8-K52.9, M09.1 |
| | | Vascular intestinal disorders | | K55-K55.9 |
| | | Gallbladder and biliary diseases | | K80-K83.9 |
| | | Pancreatitis | | K85-K86.9 |
| | | Other digestive diseases | | I84-I84.9, K20-K20.9, K22-K22.6, K22.8-K24, K31-K34, K38-K38.2, K39, K47-K49, K53-K54, K57-K62, K62.2-K62.6, K62.8-K63.4, K63.8-K64.9, K66.8, K67, K68, K69, K75.0, K77, K78-K79, K84, K87-K90.9, K92, K92.8-K93, K93.8, K96-K99 |
| | Neurological disorders | | | F00-F02.0, F02.2-F02.3, F02.8-F03.9, G10-G13.8, G20-G20.9, G23-G24, G24.1-G25.0, G25.2-G25.3, G25.5, G25.8-G26.0, G30-G31.1, G31.8-G31.9, G35-G37.9, G40-G41.9, G61-G61.9, G70-G71.1, G71.3-G72, G72.2-G73.7, G90-G90.9, G95-G95.9, M33-M33.9 |
| | | Alzheimer's disease and other dementias | | F00-F02.0, F02.8-F03.9, G30-G31.1, G31.8-G31.9 |
| | | Parkinson's disease | | F02.3, G20-G20.9 |
| | | Idiopathic epilepsy | | G40-G41.9 |
| | | Multiple sclerosis | | G35-G35.9 |
| | | Motor neuron disease | | G12.2-G12.9 |

| | | | | |
|---|---|---|---|---|
| | | Other neurological disorders | | F02.2, G10-G12.1, G13-G13.8, G23-G24, G24.1-G25.0, G25.2-G25.3, G25.5, G25.8-G26.0, G36-G37.9, G61-G61.9, G70-G71.1, G71.3-G72, G72.2-G73.7, G90-G90.9, G95-G95.9, M33-M33.9 |
| | Diabetes and kidney diseases | | | E08-E08.9, E10-E14.9, I12-I13.9, N00-N08.8, N15.0, N18-N18.9 |
| | | Diabetes mellitus | | E08-E08.1, E08.3-E08.9, E10-E10.1, E10.3-E11.1, E11.3-E14.9 |
| | | Chronic kidney disease | | E08.2, E10.2, E11.2, I12-I13.9, N02-N08.8, N15.0, N18-N18.9 |
| | | Acute glomerulonephritis | | N00-N01.9 |
| | Skin and subcutaneous diseases | | | A46-A46.0, A66-A67.3, A67.9, B86, D86.3, H05.0-H05.1, I89.1-I89.8, L00-L05.9, L08-L08.9, L10-L14.0, L51-L51.9, L88-L89.9, L97-L98.4, M72.5-M72.6 |
| | | Bacterial skin diseases | | A46-A46.0, A66-A67.3, A67.9, B86, H05.0-H05.1, I89.1-I89.8, L00-L05.9, L08-L08.9, L88, L97-L98.4, M72.5-M72.6 |
| | | Decubitus ulcer | | L89-L89.9 |
| | | Other skin and subcutaneous diseases | | D86.3, L10-L14.0, L51-L51.9 |
| | Musculoskeletal disorders | | | I27.1, L93-L93.2, M00-M03.0, M03.2-M03.6, M05-M09.0, M09.2-M09.8, M30-M32.9, M34-M36.8, M40-M43.1, M65-M65.0, M71.0-M71.1, M80-M82.8, M86.3-M86.4, M87-M87.0, M88-M89.0, M89.5, M89.7-M89.9 |
| | | Rheumatoid arthritis | | M05-M06.9, M08.0-M08.8 |

| | | | | |
|---|---|---|---|---|
| | | Other musculoskeletal disorders | | I27.1, L93-L93.2, M00-M03.0, M03.2-M03.6, M07-M08, M08.9-M09.0, M09.2-M09.8, M30-M32.9, M34-M36.8, M40-M43.1, M65-M65.0, M71.0-M71.1, M80-M82.8, M86.3-M86.4, M87-M87.0, M88-M89.0, M89.5, M89.7-M89.9 |
| | Other non-communicable diseases | | | D25-D26, D28.2, D55-D58.9, D59.1, D59.3-D59.5, D60-D61.9, D63.1, D64.0, D66-D67, D68.0-D69.4, D69.6-D69.8, D70-D70.0, D70.4-D75.8, D76-D77, D86.8, D89-D89.2, E03-E03.1, E03.3-E06.3, E06.5-E07.1, E16.1-E16.9, E20-E23.0, E23.2-E24.1, E24.3, E24.8-E27.2, E27.4-E34, E34.1-E34.8, E65-E66.0, E66.2-E68, E70-E85.2, E88-E88.2, E88.4-E88.9, G71.2, N10-N12.9, N13.6, N15, N15.1-N16.8, N20-N23.0, N25-N28.1, N29-N30.3, N30.8-N32.0, N32.3-N32.4, N34-N34.3, N36-N36.9, N39-N39.2, N41-N41.9, N44-N44.0, N45-N45.9, N49-N49.9, N72-N72.0, N75-N77.8, N80-N81.9, N83-N83.9, Q00-Q86, Q86.1-Q99.9, R95-R95.9 |
| | | Congenital birth defects | | G71.2, Q00-Q86, Q86.1-Q99.9 |
| | | | Neural tube defects | Q00-Q01.9, Q05-Q05.9 |
| | | | Congenital heart anomalies | Q20-Q28.9 |
| | | | Down syndrome | Q90-Q90.9 |
| | | | Turner syndrome | Q96-Q96.9 |

| | | | | |
|---|---|---|---|---|
| | | | Klinefelter syndrome | Q98-Q98.9 |
| | | | Other chromosomal abnormalities | Q87-Q87.8, Q91-Q93.9, Q95-Q95.9, Q97-Q97.9, Q99-Q99.8 |
| | | | Other congenital birth defects | G71.2, Q02-Q04.9, Q06-Q19, Q29-Q86, Q86.1-Q86.8, Q88-Q89.9, Q94, Q99.9 |
| | | Urinary diseases and male infertility | | N10-N12.9, N13.6, N15, N15.1-N16.8, N20-N23.0, N25-N28.1, N29-N30.3, N30.8-N32.0, N32.3-N32.4, N34-N34.3, N36-N36.9, N39-N39.2, N41-N41.9, N44-N44.0, N45-N45.9, N49-N49.9 |
| | | | Urinary tract infections and interstitial nephritis | N10-N12.9, N13.6, N15, N15.1-N16.8, N30-N30.3, N30.8-N30.9, N34-N34.3, N39.0-N39.2 |
| | | | Urolithiasis | N20-N23.0 |
| | | | Other urinary diseases | N25-N28.1, N29-N29.8, N31-N32.0, N32.3-N32.4, N36-N36.9, N39, N41-N41.9, N44-N44.0, N45-N45.9, N49-N49.9 |
| | | Gynecological diseases | | D25-D26, D28.2, E28.2, N72-N72.0, N75-N77.8, N80-N81.9, N83-N83.9 |
| | | | Uterine fibroids | D25-D26, D28.2 |
| | | | Endometriosis | N80-N80.9 |

| | | | | |
|---|---|---|---|---|
| | | | Genital prolapse | N81-N81.9 |
| | | | Other gynecological diseases | E28.2, N72-N72.0, N75-N77.8, N83-N83.9 |
| | | Hemoglobinopathies and hemolytic anemias | | D55-D58.9, D59.1, D59.3-D59.5, D60-D61.9, D63.1, D64.0 |
| | | Endocrine, metabolic, blood, and immune disorders | | D66-D67, D68.0-D69.4, D69.6-D69.8, D70-D70.0, D70.4-D75.8, D76-D77, D86.8, D89-D89.2, E03-E03.1, E03.3-E06.3, E06.5-E07.1, E16.1-E16.9, E20-E23.0, E23.2-E24.1, E24.3, E24.8-E27.2, E27.4-E28.1, E28.3-E34, E34.1-E34.8, E65-E66.0, E66.2-E68, E70-E85.2, E88-E88.2, E88.4-E88.9 |
| | | | Thyroid diseases | E03-E03.1, E03.3-E06.3, E06.5-E07, E07.1 |
| | | | Other endocrine, metabolic, blood, and immune disorders | D66-D67, D68.0-D69.4, D69.6-D69.8, D70-D70.0, D70.4-D75.8, D76-D77, D86.8, D89-D89.2, E07.0, E16.1-E16.9, E20-E23.0, E23.2-E24.1, E24.3, E24.8-E27.2, E27.4-E28.1, E28.3-E34, E34.1-E34.8, E65-E66.0, E66.2-E68, E70-E85.2, E88-E88.2, E88.4-E88.9 |
| | | Sudden infant death syndrome | | R95-R95.9 |
| | Psychiatric disorders | | | E24.4, F10-F16.9, F18-F18.9, F24, F50.0-F50.5, G31.2, G62.1, G72.1, P04.3-P04.4, Q86.0, R78.0-R78.5, X40-X46.9, X49-X49.9, X65-X65.9, Y10-Y19.9 |
| | | Mental disorders | | F24, F50.0-F50.5 |

| | | | | |
|---|---|---|---|---|
| | | | Eating disorders | F50.0-F50.5 |
| | | | Other mental disorders | F24 |
| | | Substance use disorders | | E24.4, F10-F16.9, F18-F18.9, G31.2, G62.1, G72.1, P04.3-P04.4, Q86.0, R78.0-R78.5, X40-X46.9, X49-X49.9, X65-X65.9, Y10-Y19.9 |
| | | | Alcohol use disorders | E24.4, F10-F10.9, G31.2, G62.1, G72.1, P04.3, Q86.0, R78.0, X45-X45.9, X65-X65.9, Y15-Y15.9 |
| | | | Drug use disorders | F11-F16.9, F18-F18.9, P04.4, R78.1-R78.5 |
| | | | Drug poisoning, undetermined intent | X40-X44.9, X46-X46.9, X49-X49.9, Y10-Y14.9, Y16-Y19.9 |
| | Urinary obstruction diseases | | | N09, N13-N13.5, N13.7-N13.9, N24, N28.8-N28.9, N40-N40.9 |
| Injuries | | | | D52.1, D59.0, D59.2, D59.6, D69.5, D70.1-D70.2, D78-D78.8, E03.2, E06.4, E09-E09.9, E16.0, E23.1, E24.2, E27.3, E36-E36.8, E66.1, E88.3, E89-E89.9, G21-G21.9, G24.0, G25.1, G25.4, G25.6-G25.7, G72.0, G93.7, G97-G97.9, I95.2-I95.3, I97-I97.9, I98.9, J70.0-J70.5, J95-J95.9, K43-K43.9, K52.0, K62.7, K91-K91.9, K94-K95.8, L55-L55.9, L56.3, L56.8-L56.9, L58-L58.9, M87.1, N14-N14.4, N30.4, N65-N65.1, N99-N99.9, R50.2, U00-U03, V00-W46.2, W49-W62.9, W64-W70.9, W73-W75.9, W77-W81.9, W83-W94.9, W97.9, W99-X06.9, X08-X39.9, X47-X48.9, X50-X54.9, X57-X58.9, X60- |

| | | | | |
|---|---|---|---|---|
| | | | | X64.9, X66-Y09.9, Y35-Y85.9, Y87.0-Y87.1, Y88-Y88.3, Y89.0-Y89.1 |
| | Transport injuries | | | V00-V99.0, Y85-Y85.9 |
| | | Road injuries | | V00-V04.9, V06-V80.9, V82-V82.9, V87-V88.1, V88.4-V89.9, V99-V99.0, Y85-Y85.9 |
| | | Other transport injuries | | V05-V05.9, V81-V81.9, V83-V86.9, V88.2-V88.3, V90-V98.8 |
| | Unintentional injuries | | | D52.1, D59.0, D59.2, D59.6, D69.5, D70.1-D70.2, D78-D78.8, E03.2, E06.4, E09-E09.9, E16.0, E23.1, E24.2, E27.3, E36-E36.8, E66.1, E88.3, E89-E89.9, G21-G21.9, G24.0, G25.1, G25.4, G25.6-G25.7, G72.0, G93.7, G97-G97.9, I95.2-I95.3, I97-I97.9, I98.9, J70.0-J70.5, J95-J95.9, K43-K43.9, K52.0, K62.7, K91-K91.9, K94-K95.8, L55-L55.9, L56.3, L56.8-L56.9, L58-L58.9, M87.1, N14-N14.4, N30.4, N65-N65.1, N99-N99.9, R50.2, W00-W46.2, W49-W62.9, W64-W70.9, W73-W75.9, W77-W81.9, W83-W94.9, W97.9, W99-X06.9, X08-X39.9, X47-X48.9, X50-X54.9, X57-X58.9, Y40-Y84.9, Y88-Y88.3 |
| | | Falls | | W00-W19.9 |
| | | Drowning | | W65-W70.9, W73-W74.9 |
| | | Fire, heat, and hot substances | | X00-X06.9, X08-X19.9 |
| | | Poisonings | | X47-X48.9 |

| | | | | |
|---|---|---|---|---|
| | | | Poisoning by other means | X48-X48.9 |
| | | Exposure to mechanical forces | | W20-W38.9, W40-W43.9, W45.0-W45.2, W46-W46.2, W49-W52 |
| | | Adverse effects of medical treatment | | D52.1, D59.0, D59.2, D59.6, D69.5, D70.1-D70.2, D78-D78.8, E03.2, E06.4, E09-E09.9, E16.0, E23.1, E24.2, E27.3, E36-E36.8, E66.1, E88.3, E89-E89.9, G21-G21.9, G24.0, G25.1, G25.4, G25.6-G25.7, G72.0, G93.7, G97-G97.9, I95.2-I95.3, I97-I97.9, I98.9, J70.0-J70.5, J95-J95.9, K43-K43.9, K52.0, K62.7, K91-K91.9, K94-K95.8, M87.1, N14-N14.4, N30.4, N65-N65.1, N99-N99.9, R50.2, Y40-Y84.9, Y88-Y88.3 |
| | | Animal contact | | W52.0-W62.9, W64-W64.9, X20-X29.9 |
| | | Foreign body | | W44-W45, W45.3-W45.9, W75-W75.9, W78-W80.9, W83-W84.9 |
| | | Electrocution | | W85-W87.9 |
| | | Environmental heat and cold exposure | | L55-L55.9, L56.3, L56.8-L56.9, L58-L58.9, W88-W94.9, W97.9, W99-W99.9, X30-X32.9, X39-X39.9 |
| | | Exposure to forces of nature | | X33-X38.9 |
| | | Other unintentional injuries | | W39-W39.9, W77-W77.9, W81-W81.9, X50-X54.9, X57-X58.9 |
| | Self-harm and interpersonal violence | | | U00-U03, X60-X64.9, X66-Y09.9, Y35-Y38.9, Y87.0-Y87.1, Y89.0-Y89.1 |
| | | Suicide | | X60-X64.9, X66-X84.9, Y87.0 |

| | | | | |
|---|---|---|---|---|
| | | Interpersonal violence | | X85-Y09.9, Y87.1 |
| | | Conflict and terrorism | | U00-U03, Y36-Y38.9, Y89.1 |
| | | Police conflict and executions | | Y35-Y35.9, Y89.0 |
| Garbage Code | | | | A40-A41.9, A48.0, A48.3, A49.0-A49.1, A59.0-A59.9, A61-A62, A64-A64.0, A71-A73, A74.0, A76, A99-A99.0, B07-B09, B11-B14, B16.9, B28-B29, B30-B32.4, B34-B34.1, B34.3-B36.9, B73-B74.2, B84-B85.4, B87-B88.9, B92-B94.0, B94.2-B94.9, B95.6-B95.8, C14-C14.9, C22.9, C26-C29, C35-C36, C42, C55-C55.9, C57.9, C59, C63.9, C68, C68.9, C74-C74.9, C75.9-C80.9, C87, C97-D00.0, D01, D01.4-D02, D02.4-D02.9, D07, D07.3, D07.6-D09, D09.1, D09.7, D09.9-D10, D10.9, D13, D13.9-D14, D14.4, D17-D21.9, D28, D28.9-D29, D29.9-D30, D30.9, D36.0, D36.9-D37.0, D37.6-D38, D38.6-D39.0, D39.7, D39.9-D40, D40.9-D41, D41.9, D44, D44.9, D48, D48.7-D49.1, D49.5, D49.7-D50.0, D50.9, D54, D59, D59.8-D59.9, D62-D63.0, D63.8-D64, D64.1-D65.9, D68, D69.9, D75.9, D79, D85, D87-D88, D90-D99, E07.8-E07.9, E15-E16, E17-E19, E34.0, E34.9-E35.8, E37-E39, E47-E50.9, E62, E64.1, E69, E85.3-E87.9, E90-E99.9, F04-F07.0, F07.2-F09.9, F17-F17.9, F19-F23.9, F25-F50, F50.8-G00, G00.9-G02.8, G03.9, G15-G19, G22-G22.0, G27-G29, G32-G34, G38-G39, G42-G44.8, G47-G47.2, G47.4-G60.9, G62-G62.0, G62.2-G69, G74-G89.4, G91-G93.6, G93.8-G94.8, G96-G96.9, G98-H05, H05.2-H69.9, H71-H99, I10-I10.9, I14-I15.9, I26-I27, I27.8-I27.9, I29-I29.9, I31.2-I31.4, I44-I46.9, I49-I50.9, I67.4, I70-I70.1, I70.9, I74-I76, I95-I95.1, I95.8-I95.9, J07-J08, J69-J69.9, J80-J81.1, J85-J85.3, J86-J86.9, J90-J90.0, J93-J93.1, |

| | | | | |
|---|---|---|---|---|
| | | | | J93.8-J94.9, J96-J96.9, J98.1-J98.3, K00-K19, K21-K21.9, K22.7, K30, K65-K66.1, K66.9, K68.1-K68.9, K70.4-K71.6, K71.8-K72.9, K92.0-K92.2, L06-L07, L09, L15-L50.9, L52-L54.8, L56-L56.2, L56.4-L56.5, L57-L57.9, L59-L87.9, L90-L92.9, L94-L96, L98.5-L99.8, M04, M10-M12.0, M12.2-M29, M37-M39, M43.2-M49, M49.2-M64, M65.1-M71, M71.2-M72.4, M72.8-M73, M73.8-M79.9, M83-M86.2, M86.5-M86.9, M87.2-M87.9, M89.1-M89.4, M90-M99.9, N17-N17.9, N19-N19.9, N32.1-N32.2, N32.8-N33.8, N35-N35.9, N37-N38, N39.3-N39.9, N42-N43.4, N44.1-N44.8, N46-N48.9, N50-N59, N61-N64.9, N66-N71.9, N73-N74.0, N74.2-N74.8, N78-N79, N82-N82.9, N84, N84.2-N86, N88-N95.9, N97-N97.9, R00-R19.6, R19.8-R50.1, R50.8-R78, R78.6-R94.8, R96-T98.3, U05, U08-U81, U90-U99, W47-W48, W63, W71-W72, W76-W76.9, W82, W95-W97, W98, X07, X55-X56, X59-X59.9, Y20-Y34.9, Y86-Y87, Y87.2, Y89, Y89.9-Z15.8, Z17-Z99.9 |
| | Sepsis (Non-maternal and neonatal sepsis) | | | A40-A41.9, A48.0, A48.3, A49.0-A49.1, D65-D65.9, I76, R02-R02.9, R50-R50.1, R50.8-R50.9, R56.0, R65.2 |
| | All, Ill Defined code for causes of death | | | A59.0-A59.9, A71-A71.9, A74.0, B07-B07.9, B30-B30.9, B35-B36.9, B85-B85.4, B87-B88.9, B94.0, D68, E15-E16, E50-E50.9, E64.1, F06.3-F06.4, F07.2, F09-F09.9, F30-F49, F51-F99.0, G32-G32.8, G43-G44.2, G44.4-G44.8, G47-G47.2, G47.4-G47.9, G50-G60.9, G62-G62.0, G62.2-G65.2, G89-G89.4, G99-H05, H05.2-H69.9, H71-H99, K00-K19, K30, L20-L30.9, L40-L50.9, L52-L54.8, L56-L56.2, L56.4-L56.5, L57-L57.9, L59-L68.9, L70-L76.8, L80-L87.9, L90-L92.9, L94-L96, L98.5-L99.8, M04, M10-M12.0, M12.2-M29, M37-M39, M43.2-M49, M49.2-M64, M65.1-M71, M71.2-M72.4, M72.8-M73, M73.8-M79.9, M83-M85.9, M87.2-M87.9, |

| | | | | |
|---|---|---|---|---|
| | | | | M89.1-M89.4, M90-M99.9, N32.8-N33.8, N35-N35.9, N37-N37.8, N39.3-N39.8, N42-N43.4, N44.1-N44.8, N46-N48.9, N50-N53.9, N61-N64.9, N91-N91.5, N95, N95.1-N95.9, N97-N97.9, R07.0, R08-R09, R09.3, R12-R12.0, R14-R15.9, R19-R19.6, R19.8-R23, R23.1-R30.9, R32-R39.9, R41-R49.9, R51-R53.8, R55.0, R58.0-R63.3, R63.5, R63.8, R64.0-R65.1, R66-R72.9, R74-R78, R78.6-R94.8, R96-R99.9, U05, U08-U81, U90-U99, Z00-Z15.8, Z17-Z99.9 |
| | Unspecified Infectious Diseases | | | A61-A62, A72-A73, A76, B11-B14, B28-B29, B31-B32.4, B73-B74.2, B84, B92-B94, B94.8-B94.9, B95.6-B95.8 |
| | Unspecified STD | | | A64-A64.0 |
| | Unspecified Hemorrhagic Fever | | | A99-A99.0 |
| | Unspecified Viral Diseases | | | B08-B09, B34-B34.1, B34.3-B34.9, G93.3 |
| | hepatitis B unspecified | | | B16.9 |
| | Hepatitis Unspecified | | | B94.2 |
| | Unspecified Oropharynx Cancer | | | C14-C14.9, D00.0, D10, D10.9, D37.0 |
| | Primary or secondary Liver | | | C22.9 |

| | | | | |
|---|---|---|---|---|
| | Cancer Unspecified | | | |
| | Unspecified GI Cancer | | | C26-C29, C35-C36, D00, D01, D01.4-D01.9, D13, D13.9, D37, D37.6-D37.9, D49.0 |
| | Unspecified Site Cancer | | | C42, C76, C76.7-C77, C77.3-C77.4, C77.8-C78, C79, C79.2-C80.9, C87, C97-C99, D08-D09, D09.7, D09.9, D36.0, D36.9, D48, D48.7-D49, D49.8-D49.9, D54 |
| | Unspecified Uterus Cancer | | | C55-C55.9 |
| | Unspecified Female Genital Cancer | | | C57.9, C59, D07.3, D28, D28.9, D39-D39.0, D39.7, D39.9, N84.2-N84.8 |
| | Unspecified Male Genital Cancer | | | C63.9, D07.6, D29, D29.9, D40, D40.9 |
| | Unspecified Urinary Cancer | | | C68, C68.9, D09.1, D30, D30.9, D41, D41.9 |
| | Adrenal Unspecified Site Cancer-parent cause | | | C74-C74.0 |
| | Adrenal Unspecified Site Cancer in medulla or cortex | | | C74.1 |

| | | | | |
|---|---|---|---|---|
| | Adrenal Site Cancer unspecified part of adrenal gland | | | C74.9 |
| | Unspecified Endocrine Cancer | | | C75.9, D44, D44.9, D49.7, E34.0 |
| | Head and Neck Cancer | | | C76.0-C76.1, C77.0-C77.1, C78.0-C78.3, D17-D21.9 |
| | Abdomen and Pelvis Cancer | | | C76.2-C76.3, C77.2, C77.5, C78.4-C78.8, C79.0-C79.1 |
| | upper and lower limb cancer | | | C76.4-C76.5 |
| | Unspecified Respiratory Cancer | | | D02, D02.4-D02.9, D14, D14.4, D38, D38.6, D49.1 |
| | Unspecified genital Cancer | | | D07, D49.5 |
| | Anemia Unspecified | | | D50-D50.0, D50.9, D62-D63, D63.8-D64, D64.1-D64.9, D69.9 |
| | Acquired hemolytic anemia | | | D59, D59.8-D59.9 |

| | | | | |
|---|---|---|---|---|
| | Anemia in neoplastic Diseases | | | D63.0 |
| | Unspecified Blood Diseases | | | D75.9, D79, D85, D87-D88, D90-D99 |
| | Unspecified Thyroid Diseases | | | E07.8-E07.9 |
| | Unspecified Endo/Metabolic Diseases | | | E17-E19, E35, E37-E39, E47-E49, E62, E69, E90-E99.9 |
| | Unspecified Endocrine Diseases | | | E34.9, E35.0-E35.8 |
| | Amyloidosis | | | E85.3-E85.9 |
| | Fluid, Electrolyte, Acid Base Disorders | | | E86-E87.6, E87.8-E87.9 |
| | CNS Fluid Diseases | | | E87.7 |
| | Unspecified CNS sign and symptom | | | F04-F05.9 |

| | | | | |
|---|---|---|---|---|
| | Unspecified Mental/Brain Disorders | | | F06-F06.1, F06.5-F07.0, F07.8-F08 |
| | Schizophrenia | | | F06.2, F20-F23.9, F25-F29.9 |
| | Assigned death to tobacco | | | F17-F17.9 |
| | Undetermined intent Poisoning by multiple or unspecified drug | | | F19-F19.9 |
| | Unspecified Eating Disorders | | | F50, F50.8-F50.9 |
| | Unspecified Meningitis | | | G00, G00.9-G02.8, G03.9 |
| | Unspecified CNS Diseases | | | G15-G19, G22-G22.0, G27-G29, G33-G34, G38-G39, G42, G48-G49, G66-G69, G74-G79, G84-G88 |
| | External Causes UDI, type unspecified | | | G44.3, G91.3, R58, S00-T98.3, Y24.5-Y24.7, Y25.2, Y26.3, Y27.4-Y27.5, Y28.3, Y28.5, Y29.3, Y33-Y34.9, Y86-Y87, Y87.2, Y89, Y89.9-Y99.9 |
| | Cerebral Palsy | | | G80-G80.9, G82.1, G82.4, G83.0, G83.8 |
| | Plegia | | | G81-G82.0, G82.2-G82.3, G82.5-G83, G83.1-G83.5, G83.9 |

| | | | | |
|---|---|---|---|---|
| | Intermediate cause for CNS | | | G91-G91.2, G91.4-G93, G93.1-G93.2, G93.4-G93.6, G94.0-G94.8 |
| | Cerebral Cysts | | | G93.0 |
| | Unspecified Brain Diseases | | | G93.8-G94, G96-G96.9, G98-G98.9 |
| | Hypertension | | | I10-I10.9, I15-I15.9, I67.4, R03-R03.0, R04.0 |
| | Unspecified cardiovascular diseases | | | I14, I29-I29.9 |
| | Pulmonary Embolism | | | I26-I26.9 |
| | right heart failure and pulmonary heart disease | | | I27, I27.8-I27.9 |
| | Pneumothorax | | | I31.2-I31.4, J93-J93.1, J93.8-J93.9, J94.2, R04, R04.1-R04.9 |
| | Cardiac rhythm disorders | | | I44-I45.9, I49-I49.9 |
| | Shock, Cardiac Arrest, Coma | | | I46-I46.9, I95-I95.1, I95.8-I95.9, R03.1, R09.0, R09.2, R09.8, R40-R40.4, R55, R56, R56.1-R57.9 |
| | Heart failure unspecified right or left | | | I50, I50.8-I50.9, J81, J81.1 |
| | Left heart failure | | | I50.0-I50.4 |

| | | | | |
|---|---|---|---|---|
| | Atherosclerosis | | | I70-I70.1, I70.9 |
| | Arterial Embolism | | | I74-I75.8 |
| | Unspecified acute respiratory infectious | | | J07-J08 |
| | Pneumonitis | | | J69-J69.9, J85-J85.3 |
| | Acute Respiratory Failure | | | J80-J80.9, J81.0, J96-J96.0, J96.9, J98.1-J98.3 |
| | Pleurisy, Pyothorax | | | J86-J86.9, J90-J90.0, J94-J94.1, J94.8-J94.9, R09.1 |
| | Chronic respiratory failure | | | J96.1-J96.8 |
| | Unspecified Intestine Diseases | | | K21-K21.9, K22.7 |
| | Peritonitis & Acute Abdomen | | | K65-K66.1, K66.9, K68.1-K68.9, R10-R10.9 |
| | Alcoholic hepatic failure | | | K70.4-K70.9 |
| | Hepatic Failure | | | K71-K71.6, K71.8-K72.9, R16-R18.9 |

| | | | | |
|---|---|---|---|---|
| | Gastrointestinal Bleeding | | | K92.0-K92.2 |
| | Unspecified Skin Diseases | | | L06-L07, L09, L15-L19, L31-L39, L69, L77-L79 |
| | Osteomyelitis | | | M86-M86.2, M86.5-M86.9 |
| | Acute kidney failure | | | N17-N17.9, N19-N19.9 |
| | Fistula | | | N32.1-N32.2, N82-N82.9 |
| | Unspecified Urinary Diseases | | | N38, N39.9, N54-N59, N66-N69, N78-N79, N84, N84.9-N86, N88-N90.9 |
| | Female pelvic inflammatory diseases | | | N70-N71.9, N73-N74.0, N74.2-N74.8 |
| | Unspecified Gynecologic Diseases | | | N92-N94.9, N95.0 |
| | Unspecified sign and symptom for Heart diseases | | | R00-R01.2, R07, R07.1-R07.9 |
| | Unspecified sign and symptom for Respiratory diseases | | | R05-R06.9, R23.0 |

| | | | | |
|---|---|---|---|---|
| | Nausea and Vomiting | | | R11-R11.9 |
| | Unspecified sign and symptom for GI diseases | | | R13-R13.9 |
| | Urinary Obstruction Diseases | | | R31-R31.9 |
| | Senility | | | R54-R54.9 |
| | Cachexia | | | R63.4, R63.6, R64 |
| | Diabetes unspecified type | | | R73-R73.9 |
| | Exposure to unspecified factor X59 | | | W47-W48, W63, W71-W72, W82, W95-W97, W98, X07, X55-X56, X59-X59.9 |
| | Undetermined intent Strangulation | | | W76-W76.9, Y20-Y20.9 |
| | Undetermined intent Drowning | | | Y21-Y21.9 |
| | Undetermined intent shooting | | | Y22-Y22.9 |

| | | | | |
|---|---|---|---|---|
| | by Handgun Firearm | | | |
| | Undetermined intent shooting by rifle and larger firearm | | | Y23-Y23.2, Y23.4-Y23.7 |
| | Undetermined intent shooting by unspecified firearm | | | Y23.3, Y23.8-Y24.4, Y24.8-Y24.9 |
| | Undetermined intent of Explosion | | | Y25-Y25.1, Y25.4-Y25.9 |
| | Undetermined intent of fire and flames | | | Y26-Y26.2, Y26.4-Y26.9 |
| | Undetermined intent of Hot Objects | | | Y27-Y27.3, Y27.6-Y27.9 |
| | Undetermined intent of Sharp Objects | | | Y28-Y28.2, Y28.4, Y28.6-Y28.9 |
| | Undetermined intent of Blunt Objects | | | Y29-Y29.0 |
| | Undetermined intent of fall | | | Y29.1-Y29.2, Y29.4-Y30.9 |

| | | | | |
|---|---|---|---|---|
| | Undetermined intent of Moving Objects | | | Y31-Y31.9 |
| | Undetermined intent of Crashing | | | Y32-Y32.9 |
| Still Born | | | | P95-P95.9 |

# Supplemental Figures

## Results for Depressive Disorders, Bipolar Disorder, and Schizophrenia

For each patient and non-fatal diagnosis (Depressive Disorders, Bipolar Disorder, and Schizophrenia), we analysed the mortality outcomes at any point during the remaining observation period after three months and used a cutoff point of at least 100 cases from each cause of death.

Appendix figure 1: Significant relative risk of mortality and excess mortality rate for all ages by sex with depressive disorder exposure longer than three months. Causes of death with fewer than 100 cases in the given age and sex are excluded.

| | Relative Risk | | | Excess Mortality Rate | | |
|---|---|---|---|---|---|---|
| | All sexes | Male | Female | All sexes | Male | Female |
| Suicide | 17.92 (17.03, 18.86) | 16.41 (15.35, 17.54) | 26.62 (24.59, 28.82) | 0.25 | 0.33 | 0.19 |
| Idiopathic epilepsy | 3.59 (2.96, 4.36) | | | 0.01 | | |
| Alcohol use disorders | 3.51 (3.13, 3.93) | 3.9 (3.45, 4.42) | | 0.04 | 0.08 | |
| Drowning | 3.01 (2.49, 3.64) | | | 0.01 | | |
| Road injuries | 2.11 (1.94, 2.29) | 2.31 (2.08, 2.55) | 2.38 (2.03, 2.78) | 0.05 | 0.09 | 0.03 |
| Alzheimer's disease and other dementias | 2.08 (1.83, 2.36) | | 2.02 (1.72, 2.36) | 0.02 | | 0.02 |
| HIV/AIDS | 2.06 (1.86, 2.28) | 1.99 (1.73, 2.3) | 2.37 (2.05, 2.74) | 0.03 | 0.04 | 0.03 |
| Interpersonal violence | 1.78 (1.64, 1.93) | 1.88 (1.71, 2.07) | 2.6 (2.21, 3.07) | 0.04 | 0.08 | 0.03 |
| Other endocrine, metabolic, blood, and immune disorders | 1.76 (1.48, 2.08) | | | 0.01 | | |
| Cirrhosis and other chronic liver diseases | 1.73 (1.6, 1.88) | 2.06 (1.88, 2.26) | 1.5 (1.27, 1.77) | 0.04 | 0.1 | 0.01 |
| Falls | 1.43 (1.24, 1.65) | 1.73 (1.43, 2.09) | | 0.01 | 0.02 | |
| Lower respiratory tract diseases | 1.29 (1.21, 1.37) | 1.44 (1.31, 1.57) | 1.2 (1.1, 1.31) | 0.04 | 0.05 | 0.02 |
| Ischemic heart disease | 1.11 (1.05, 1.17) | 1.15 (1.07, 1.24) | 1.13 (1.06, 1.22) | 0.03 | 0.04 | 0.03 |
| Urinary tract infections and interstitial nephritis | | | 1.33 (1.11, 1.6) | | | 0.01 |

Highest Risk

Lowest Risk

Appendix figure 2: Significant relative risk of mortality and excess mortality rate for all sexes by age with depressive disorder exposure longer than three months. Causes of death with fewer than 100 cases in the given age and sex are excluded.

| | Relative Risk | | | | Excess Mortality Rate | | | |
|---|---|---|---|---|---|---|---|---|
| | All Ages | 15 to 29 | 30 to 59 | 60 plus | All Ages | 15 to 29 | 30 to 59 | 60 plus |
| Suicide | 17.92 (17.03, 18.86) | 14.09 (12.65, 15.71) | 12.66 (11.91, 13.47) | 16.86 (14.13, 20.12) | 0.25 | 0.2 | 0.5 | 0.15 |
| Idiopathic epilepsy | 3.59 (2.96, 4.36) | | | | 0.01 | | | |
| Alcohol use disorders | 3.51 (3.13, 3.93) | | 2.04 (1.81, 2.31) | | 0.04 | | 0.07 | |
| Drowning | 3.01 (2.49, 3.64) | | | | 0.01 | | | |
| Road injuries | 2.11 (1.94, 2.29) | 2.03 (1.72, 2.4) | 1.62 (1.46, 1.8) | | 0.05 | 0.05 | 0.07 | |
| Alzheimer's disease and other dementias | 2.08 (1.83, 2.36) | | | 3.24 (2.81, 3.73) | 0.02 | | | 0.17 |
| HIV/AIDS | 2.06 (1.86, 2.28) | | 1.22 (1.08, 1.36) | | 0.03 | | 0.03 | |
| Interpersonal violence | 1.78 (1.64, 1.93) | 1.51 (1.32, 1.72) | 1.57 (1.41, 1.74) | | 0.04 | 0.05 | 0.06 | |
| Other endocrine, metabolic, blood, and immune disorders | 1.76 (1.48, 2.08) | | | | 0.01 | | | |
| Cirrhosis and other chronic liver diseases | 1.73 (1.6, 1.88) | | 1.18 (1.08, 1.29) | | 0.04 | | 0.04 | |
| Falls | 1.43 (1.24, 1.65) | | 1.73 (1.44, 2.09) | | 0.01 | | 0.02 | |
| Lower respiratory tract diseases | 1.29 (1.21, 1.37) | | 1.78 (1.63, 1.95) | 1.34 (1.21, 1.47) | 0.04 | | 0.11 | 0.13 |
| Ischemic heart disease | 1.11 (1.05, 1.17) | | 1.21 (1.13, 1.29) | | 0.03 | | 0.08 | |
| Chronic obstructive pulmonary disease | | | 1.36 (1.21, 1.52) | | | | 0.04 | |
| Stroke | | | 1.13 (1.05, 1.21) | | | | 0.04 | |

Highest Risk

Lowest Risk

Appendix figure 3: Significant relative risk of mortality and excess mortality rate for all ages by sex with bipolar disorder exposure longer than three months. Causes of death with fewer than 100 cases in the given age and sex are excluded.

| | Relative Risk | | | Excess Mortality Rate | | |
|---|---|---|---|---|---|---|
| | All sexes | Male | Female | All sexes | Male | Female |
| Suicide | 11.25 (10.61, 11.93) | 9.88 (9.12, 10.72) | 18.19 (16.68, 19.82) | 0.18 | 0.22 | 0.15 |
| Drowning | 4.07 (3.5, 4.74) | 4.11 (3.42, 4.95) | | 0.02 | 0.03 | |
| Idiopathic epilepsy | 3.41 (2.84, 4.09) | | | 0.01 | | |
| Other endocrine, metabolic, blood, and immune disorders | 2.65 (2.33, 3.01) | | 3.21 (2.77, 3.71) | 0.03 | | 0.04 |
| Road injuries | 1.93 (1.78, 2.1) | 1.94 (1.75, 2.15) | 2.78 (2.43, 3.17) | 0.05 | 0.07 | 0.04 |
| HIV/AIDS | 1.9 (1.72, 2.09) | 1.67 (1.44, 1.94) | 2.42 (2.12, 2.75) | 0.03 | 0.03 | 0.04 |
| Interpersonal violence | 1.77 (1.64, 1.91) | 1.92 (1.76, 2.1) | 2.74 (2.37, 3.17) | 0.05 | 0.1 | 0.03 |
| Alcohol use disorders | 1.46 (1.24, 1.71) | 1.5 (1.24, 1.81) | | 0.01 | 0.01 | |
| Other cardiovascular and circulatory diseases | 1.41 (1.23, 1.61) | | 1.66 (1.4, 1.95) | 0.01 | | 0.02 |
| Urinary tract infections and interstitial nephritis | 1.39 (1.22, 1.59) | | 1.65 (1.42, 1.92) | 0.01 | | 0.02 |
| Falls | 1.36 (1.19, 1.56) | 1.49 (1.23, 1.81) | 1.36 (1.12, 1.65) | 0.01 | 0.01 | 0.01 |
| Lower respiratory tract diseases | 1.31 (1.24, 1.39) | 1.31 (1.2, 1.44) | 1.36 (1.26, 1.46) | 0.05 | 0.04 | 0.05 |
| Ischemic heart disease | 1.26 (1.21, 1.32) | 1.18 (1.11, 1.27) | 1.43 (1.35, 1.51) | 0.07 | 0.05 | 0.11 |
| Diabetes mellitus | 1.1 (1.04, 1.17) | | 1.19 (1.1, 1.28) | 0.02 | | 0.04 |
| Cardiomyopathy and myocarditis | | | 1.25 (1.07, 1.45) | | | 0.01 |

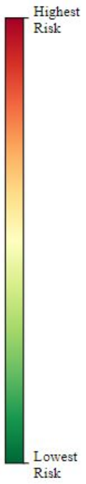

Appendix figure 4: Significant relative risk of mortality and excess mortality rate for all sexes by age with bipolar disorder exposure longer than three months. Causes of death with fewer than 100 cases in the given age and sex are excluded.

| | Relative Risk | | | | Excess Mortality Rate | | | |
|---|---|---|---|---|---|---|---|---|
| | All Ages | 15 to 29 | 30 to 59 | 60 plus | All Ages | 15 to 29 | 30 to 59 | 60 plus |
| Suicide | 11.25 (10.61, 11.93) | 10.75 (9.62, 12.02) | 7.47 (6.95, 8.03) | | 0.18 | 0.19 | 0.34 | |
| Drowning | 4.07 (3.5, 4.74) | | 3.56 (2.96, 4.28) | | 0.02 | | 0.04 | |
| Idiopathic epilepsy | 3.41 (2.84, 4.09) | | | | 0.01 | | | |
| Other endocrine, metabolic, blood, and immune disorders | 2.65 (2.33, 3.01) | | 2.65 (2.29, 3.07) | | 0.03 | | 0.06 | |
| Road injuries | 1.93 (1.78, 2.1) | 1.83 (1.56, 2.14) | 1.43 (1.29, 1.58) | | 0.05 | 0.05 | 0.06 | |
| HIV/AIDS | 1.9 (1.72, 2.09) | | | | 0.03 | | | |
| Interpersonal violence | 1.77 (1.64, 1.91) | 1.63 (1.45, 1.82) | 1.39 (1.26, 1.55) | | 0.05 | 0.08 | 0.05 | |
| Alcohol use disorders | 1.46 (1.24, 1.71) | | | | 0.01 | | | |
| Other cardiovascular and circulatory diseases | 1.41 (1.23, 1.61) | | 1.86 (1.59, 2.18) | | 0.01 | | 0.04 | |
| Urinary tract infections and interstitial nephritis | 1.39 (1.22, 1.59) | | 2.53 (2.14, 3.0) | | 0.01 | | 0.04 | |
| Falls | 1.36 (1.19, 1.56) | | 1.56 (1.3, 1.86) | | 0.01 | | 0.02 | |
| Lower respiratory tract diseases | 1.31 (1.24, 1.39) | | 2.16 (2.01, 2.33) | 1.27 (1.14, 1.41) | 0.05 | | 0.19 | 0.1 |
| Ischemic heart disease | 1.26 (1.21, 1.32) | | 1.56 (1.48, 1.64) | | 0.07 | | 0.27 | |
| Diabetes mellitus | 1.1 (1.04, 1.17) | | 1.37 (1.28, 1.48) | | 0.02 | | 0.1 | |
| Cardiomyopathy and myocarditis | | | 1.23 (1.07, 1.41) | | | | 0.02 | |
| Chronic obstructive pulmonary disease | | | 1.63 (1.48, 1.8) | | | | 0.08 | |
| Hypertensive heart disease | | | 1.48 (1.3, 1.69) | | | | 0.04 | |
| Other chronic respiratory diseases | | | 1.6 (1.32, 1.93) | | | | 0.02 | |
| Stroke | | | 1.18 (1.11, 1.26) | | | | 0.07 | |

Highest Risk

Lowest Risk

Appendix figure 5: Significant relative risk of mortality and excess mortality rate for all ages by sex with schizophrenia exposure longer than three months. Causes of death with fewer than 100 cases in the given age and sex are excluded.

| | Relative Risk | | | Excess Mortality Rate | | |
|---|---|---|---|---|---|---|
| | All sexes | Male | Female | All sexes | Male | Female |
| Suicide | 10.37 (9.95, 10.81) | 7.53 (7.18, 7.91) | 14.53 (13.4, 15.75) | 0.37 | 0.61 | 0.18 |
| Drug poisoning, undetermined intent | 8.92 (7.64, 10.41) | 6.97 (5.75, 8.44) | | 0.03 | 0.04 | |
| Idiopathic epilepsy | 7.23 (6.64, 7.87) | 5.33 (4.8, 5.92) | 9.62 (8.32, 11.13) | 0.08 | 0.12 | 0.05 |
| Drowning | 6.44 (5.94, 6.98) | 4.3 (3.92, 4.72) | 12.71 (10.75, 15.03) | 0.09 | 0.14 | 0.04 |
| Fire, heat, and hot substances | 6.33 (5.43, 7.37) | | | 0.03 | | |
| Foreign body | 5.81 (5.18, 6.51) | 5.16 (4.49, 5.92) | | 0.04 | 0.07 | |
| Alcohol use disorders | 3.21 (2.99, 3.45) | 2.37 (2.19, 2.56) | | 0.09 | 0.16 | |
| Tuberculosis | 3.18 (2.93, 3.44) | 2.64 (2.42, 2.89) | 2.88 (2.41, 3.45) | 0.07 | 0.12 | 0.02 |
| Interpersonal violence | 2.86 (2.75, 2.97) | 2.18 (2.09, 2.27) | 3.03 (2.7, 3.4) | 0.28 | 0.49 | 0.06 |
| Road injuries | 2.8 (2.68, 2.93) | 2.1 (2.0, 2.21) | 3.55 (3.22, 3.91) | 0.21 | 0.32 | 0.09 |
| Other endocrine, metabolic, blood, and immune disorders | 2.65 (2.44, 2.89) | 1.93 (1.71, 2.19) | 3.64 (3.24, 4.08) | 0.06 | 0.05 | 0.06 |
| HIV/AIDS | 2.09 (1.96, 2.22) | 1.59 (1.47, 1.72) | 2.67 (2.41, 2.96) | 0.09 | 0.1 | 0.07 |
| Falls | 2.01 (1.87, 2.17) | 1.82 (1.66, 1.99) | 1.88 (1.64, 2.16) | 0.06 | 0.09 | 0.03 |
| Pancreatitis | 1.99 (1.77, 2.24) | 1.65 (1.44, 1.9) | | 0.02 | 0.03 | |
| Upper digestive system diseases | 1.97 (1.74, 2.22) | 1.67 (1.44, 1.93) | | 0.02 | 0.03 | |
| Protein-energy malnutrition | 1.92 (1.76, 2.1) | 1.74 (1.56, 1.93) | 1.9 (1.64, 2.21) | 0.04 | 0.06 | 0.02 |
| Paralytic ileus and intestinal obstruction | 1.92 (1.7, 2.16) | 1.63 (1.38, 1.91) | 2.24 (1.88, 2.68) | 0.02 | 0.02 | 0.02 |
| Diarrheal diseases | 1.73 (1.52, 1.95) | 1.45 (1.22, 1.71) | 2.05 (1.71, 2.46) | 0.02 | 0.02 | 0.02 |
| Lower respiratory tract diseases | 1.7 (1.64, 1.76) | 1.48 (1.41, 1.54) | 1.86 (1.76, 1.96) | 0.24 | 0.27 | 0.18 |
| Cirrhosis and other chronic liver diseases | 1.63 (1.55, 1.71) | 1.29 (1.22, 1.37) | 1.54 (1.36, 1.75) | 0.1 | 0.12 | 0.03 |
| Ischemic heart disease | 1.61 (1.57, 1.65) | 1.28 (1.24, 1.32) | 1.95 (1.87, 2.03) | 0.39 | 0.32 | 0.34 |
| Other chronic respiratory diseases | 1.59 (1.46, 1.74) | 1.24 (1.1, 1.4) | 2.02 (1.77, 2.31) | 0.03 | 0.02 | 0.03 |
| Other cardiovascular and circulatory diseases | 1.58 (1.45, 1.72) | 1.21 (1.08, 1.36) | 2.04 (1.8, 2.31) | 0.03 | 0.02 | 0.04 |
| Cardiomyopathy and myocarditis | 1.44 (1.35, 1.54) | 1.16 (1.07, 1.26) | 1.7 (1.52, 1.89) | 0.05 | 0.03 | 0.04 |
| Other digestive diseases | 1.43 (1.26, 1.63) | 1.26 (1.07, 1.49) | | 0.01 | 0.01 | |
| Acute hepatitis | 1.39 (1.17, 1.65) | | | 0.01 | | |
| Urinary tract infections and interstitial nephritis | 1.34 (1.22, 1.46) | | 1.8 (1.6, 2.02) | 0.02 | | 0.04 |
| Vascular intestinal disorders | 1.26 (1.06, 1.49) | | | 0.0 | | |
| Asthma | 1.22 (1.02, 1.46) | | | 0.0 | | |
| Diabetes mellitus | 1.19 (1.15, 1.24) | | 1.5 (1.42, 1.58) | 0.07 | | 0.14 |
| Chronic kidney disease | | | 1.14 (1.02, 1.27) | | | 0.01 |
| Chronic obstructive pulmonary disease | | | 1.25 (1.16, 1.35) | | | 0.04 |
| Hypertensive heart disease | | | 1.16 (1.05, 1.28) | | | 0.02 |
| Stroke | | | 1.26 (1.2, 1.32) | | | 0.11 |

Highest Risk

Lowest Risk

Appendix figure 6: Significant relative risk of mortality and excess mortality rate for all sexes by age with schizophrenia exposure longer than three months. Causes of death with fewer than 100 cases in the given age and sex are excluded.

| | Relative Risk | | | | Excess Mortality Rate | | | |
|---|---|---|---|---|---|---|---|---|
| | All Ages | 15 to 29 | 30 to 59 | 60 plus | All Ages | 15 to 29 | 30 to 59 | 60 plus |
| Suicide | 10.37 (9.95, 10.81) | 11.72 (10.94, 12.56) | 6.51 (6.17, 6.87) | | 0.37 | 0.51 | 0.62 | |
| Drug poisoning, undetermined intent | 8.92 (7.64, 10.41) | | | | 0.03 | | | |
| Idiopathic epilepsy | 7.23 (6.64, 7.87) | 6.82 (5.62, 8.28) | 5.8 (5.25, 6.42) | | 0.08 | 0.06 | 0.17 | |
| Drowning | 6.44 (5.94, 6.98) | 9.13 (8.01, 10.42) | 5.1 (4.58, 5.68) | | 0.09 | 0.13 | 0.15 | |
| Fire, heat, and hot substances | 6.33 (5.43, 7.37) | | 6.47 (5.4, 7.76) | | 0.03 | | 0.05 | |
| Foreign body | 5.81 (5.18, 6.51) | | 8.47 (7.37, 9.74) | | 0.04 | | 0.1 | |
| Alcohol use disorders | 3.21 (2.99, 3.45) | | 1.87 (1.73, 2.02) | | 0.09 | | 0.15 | |
| Tuberculosis | 3.18 (2.93, 3.44) | 5.7 (4.75, 6.84) | 2.35 (2.14, 2.58) | | 0.07 | 0.06 | 0.13 | |
| Interpersonal violence | 2.86 (2.75, 2.97) | 2.6 (2.46, 2.75) | 2.17 (2.05, 2.29) | | 0.28 | 0.5 | 0.33 | |
| Road injuries | 2.8 (2.68, 2.93) | 2.82 (2.61, 3.05) | 2.08 (1.96, 2.2) | | 0.21 | 0.26 | 0.31 | |
| Other endocrine, metabolic, blood, and immune disorders | 2.65 (2.44, 2.89) | | 2.74 (2.48, 3.03) | | 0.06 | | 0.13 | |
| HIV/AIDS | 2.09 (1.96, 2.22) | 2.1 (1.87, 2.36) | 1.12 (1.05, 1.21) | | 0.09 | 0.1 | 0.04 | |
| Falls | 2.01 (1.87, 2.17) | | 2.7 (2.46, 2.96) | 1.48 (1.26, 1.74) | 0.06 | | 0.15 | 0.06 |
| Pancreatitis | 1.99 (1.77, 2.24) | | 1.58 (1.38, 1.82) | | 0.02 | | 0.04 | |
| Upper digestive system diseases | 1.97 (1.74, 2.22) | | 2.57 (2.23, 2.95) | | 0.02 | | 0.06 | |
| Protein-energy malnutrition | 1.92 (1.76, 2.1) | | 4.32 (3.86, 4.83) | 1.83 (1.56, 2.13) | 0.04 | | 0.13 | 0.09 |
| Paralytic ileus and intestinal obstruction | 1.92 (1.7, 2.16) | | 3.29 (2.85, 3.8) | | 0.02 | | 0.07 | |
| Diarrheal diseases | 1.73 (1.52, 1.95) | | 3.72 (3.2, 4.34) | | 0.02 | | 0.06 | |
| Lower respiratory tract diseases | 1.7 (1.64, 1.76) | 4.75 (4.23, 5.33) | 3.05 (2.92, 3.18) | 1.47 (1.38, 1.57) | 0.24 | 0.15 | 0.73 | 0.37 |
| Cirrhosis and other chronic liver diseases | 1.63 (1.55, 1.71) | 3.05 (2.62, 3.56) | 1.1 (1.04, 1.16) | | 0.1 | 0.07 | 0.06 | |
| Ischemic heart disease | 1.61 (1.57, 1.65) | 7.09 (6.34, 7.94) | 2.21 (2.15, 2.27) | | 0.39 | 0.18 | 1.27 | |
| Other chronic respiratory diseases | 1.59 (1.46, 1.74) | | 2.35 (2.11, 2.62) | | 0.03 | | 0.1 | |
| Other cardiovascular and circulatory diseases | 1.58 (1.45, 1.72) | | 2.12 (1.92, 2.34) | | 0.03 | | 0.11 | |
| Cardiomyopathy and myocarditis | 1.44 (1.35, 1.54) | | 1.73 (1.6, 1.87) | | 0.05 | | 0.14 | |
| Other digestive diseases | 1.43 (1.26, 1.63) | | 1.95 (1.67, 2.28) | | 0.01 | | 0.04 | |
| Acute hepatitis | 1.39 (1.17, 1.65) | | | | 0.01 | | | |
| Urinary tract infections and interstitial nephritis | 1.34 (1.22, 1.46) | | 2.61 (2.33, 2.93) | 1.2 (1.02, 1.41) | 0.02 | | 0.1 | 0.03 |
| Vascular intestinal disorders | 1.26 (1.06, 1.49) | | | | 0.0 | | | |
| Asthma | 1.22 (1.02, 1.46) | | | | 0.0 | | | |
| Diabetes mellitus | 1.19 (1.15, 1.24) | 4.19 (3.56, 4.94) | 1.6 (1.53, 1.67) | | 0.07 | 0.07 | 0.35 | |
| Alzheimer's disease and other dementias | | | 4.36 (3.59, 5.29) | 1.28 (1.1, 1.5) | | | 0.04 | 0.04 |
| Chronic kidney disease | | | 1.1 (1.01, 1.2) | | | | 0.02 | |
| Chronic obstructive pulmonary disease | | | 2.03 (1.92, 2.15) | | | | 0.3 | |
| Gallbladder and biliary diseases | | | 1.74 (1.47, 2.06) | | | | 0.03 | |
| Hypertensive heart disease | | | 1.95 (1.8, 2.11) | | | | 0.15 | |
| Stroke | | 3.12 (2.67, 3.65) | 1.58 (1.52, 1.64) | | | 0.07 | 0.49 | |

Highest Risk

Lowest Risk

# GATHER Compliance: Guidelines for Accurate and Transparent Health Estimates Reporting

Checklist of information that should be included in new reports of global health estimates

| Item # | Checklist item | Reported on page # |
|---|---|---|
| Objectives and funding | | |
| 1 | Define the indicator(s), populations (including age, sex, and geographic entities), and time period(s) for which estimates were made. | Main text methods paragraph, "Design, setting, and participants" |
| 2 | List the funding sources for the work. | Main text acknowledgements section |
| Data Inputs | | |
| *For all data inputs from multiple sources that are synthesized as part of the study:* | | |
| 3 | Describe how the data were identified and how the data were accessed. | Main text methods paragraph, "Data sources" |
| 4 | Specify the inclusion and exclusion criteria. Identify all ad-hoc exclusions. | Supplementary Appendix pp 4 |
| 5 | Provide information on all included data sources and their main characteristics. For each data source used, report reference information or contact name/institution, population represented, data collection method, year(s) of data collection, sex and age range, diagnostic criteria or measurement method, and sample size, as relevant. | Main text methods paragraph, "Data sources" |
| 6 | Identify and describe any categories of input data that have potentially important biases (e.g., based on characteristics listed in item 5). | Main text methods paragraph, "Data sources", Main text "Limitations", Supplementary Appendix pp 3-5 |
| *For data inputs that contribute to the analysis but were not synthesized as part of the study:* | | |
| 7 | Describe and give sources for any other data inputs. | n/a |
| *For all data inputs:* | | |
| 8 | Provide all data inputs in a file format from which data can be efficiently extracted (e.g., a spreadsheet rather than a PDF), including all relevant meta-data listed in item 5. For any data inputs that cannot be shared because of ethical or legal reasons, such as third-party ownership, provide a contact name or the name of the institution that retains the right to the data. | Spreadsheet of data inputs available from the first and corresponding authors upon request. |
| Data analysis | | |

| | | |
|---|---|---|
| 9 | Provide a conceptual overview of the data analysis method. A diagram may be helpful. | Supplementary Appendix pp 3-5 |
| 10 | Provide a detailed description of all steps of the analysis, including mathematical formulae. This description should cover, as relevant, data cleaning, data pre-processing, data adjustments and weighting of data sources, and mathematical or statistical model(s). | Supplementary Appendix pp 3-5 |
| 11 | Describe how candidate models were evaluated and how the final model(s) were selected. | n/a |
| 12 | Provide the results of an evaluation of model performance, if done, as well as the results of any relevant sensitivity analysis. | n/a |
| 13 | Describe methods for calculating uncertainty of the estimates. State which sources of uncertainty were, and were not, accounted for in the uncertainty analysis. | Supplementary Appendix pp 5 |
| 14 | State how analytic or statistical source code used to generate estimates can be accessed. | GitHub URL will be provided at the time of publication |
| Results and Discussion | | |
| 15 | Provide published estimates in a file format from which data can be efficiently extracted. | CSV files available upon request to the corresponding author |
| 16 | Report a quantitative measure of the uncertainty of the estimates (e.g. uncertainty intervals). | Confidence intervals are given for all findings, including in the main text, figures, and tables. |
| 17 | Interpret results in light of existing evidence. If updating a previous set of estimates, describe the reasons for changes in estimates. | Main text Research in Context section "Implications of all the available evidence" and main text Discussion section pp 8-11 |
| 18 | Discuss limitations of the estimates. Include a discussion of any modelling assumptions or data limitations that affect interpretation of the estimates. | Main text limitations section |

*This checklist should be used in conjunction with the GATHER statement and Explanation and Elaboration document, found on gather-statement.org*
